# Cold seep nitrogen fixation and its potential relationship with sulfur cycling

**DOI:** 10.1128/spectrum.00536-24

**Published:** 2024-08-22

**Authors:** Qiumei Quan, Jiaxing Liu, Xiaomin Xia, Si Zhang, Zhixin Ke, Minxiao Wang, Yehui Tan

**Affiliations:** 1 South China Sea Institute of Oceanology, Chinese Academy of Sciences, Guangzhou, China; 2 University of Chinese Academy of Sciences, Beijing, China; 3 Southern Marine Science and Engineering Guangdong Laboratory (Guangzhou), Guangzhou, China; 4 Center of Deep-Sea Research, Institute of Oceanology, Chinese Academy of Sciences, Qingdao, China; University of Mississippi, University, Mississippi, USA

**Keywords:** diazotroph, biogeochemical cycling, cold seep, dissolved organic matter, *Dechloromonas *genus

## Abstract

Dinitrogen (N_2_) fixation is a crucial source of bioavailable nitrogen in carbon-dominated cold seep systems. Previous studies have shown that diazotrophy is not necessarily dependent on sulfate-dependent anaerobic oxidation of methane for energy, and diverse catabolism can fuel the high-energy-demanding process in sediments. However, it remains unclear whether diazotroph can obtain energy by sulfur oxidation in sulfur-rich cold seep water column. Here, field investigations and *in situ* experiments were conducted in Haima cold seep to examine the effects of diverse sources of dissolved organic matter (DOM) on N_2_ fixation, specifically containing sulfur, carbon, nitrogen, and phosphorus. We found that active N_2_ fixation occurred in the water column above the Haima cold seep, with the *Dechloromonas* genus dominating the diazotroph community as revealed by *nifH* gene using high-throughput sequencing. *In situ* experiments showed an increased rate of N_2_ fixation (1.15- to 12.70-fold compared to that in control group) and a greater relative abundance of the *Dechloromonas* genus following enrichment with sulfur-containing organic matter. Furthermore, metagenomic assembly and binning revealed that *Dechloromonas* sp. carried genes related to N_2_ fixation (*nifDHK*) and sulfur compound oxidation (*fccAB* and *soxABCXYZ*), implying that the genus potentially serves as a multifunctional mediator for N_2_ fixation and sulfur cycling. Our results provide new insights regarding potential coupling mechanism associated with sulfur-driven N_2_ fixation in methane- and sulfide-rich environments.

**IMPORTANCE:**

N2 fixation is an important source of biologically available in carbon-dominated cold seep systems as little nitrogen is released by hydrocarbon seepage, thereby promoting biological productivity and the degradation of non-nitrogenous organic matter. Cold seeps are rich in diverse sources of dissolved organic matter (DOM) derived from the sinking of photosynthetic products in euphotic layer and the release of chemosynthesis products on the seafloor. However, it remains unclear whether N2 fixation is coupled to the metabolic processes of DOM, as determined by e.g., carbon, nitrogen, phosphorus, and sulfur content, for energy acquisition in sulfur-rich cold seeps. In this study, diazotroph community structure and its response to DOM compositions were revealed. Moreover, the metagenomics analysis suggested that *Dechloromonas* genus plays a dominant role in potential coupling N2 fixation and sulfur oxidation. Our study highlighted that sulfur oxidation in deep-sea cold seeps may serve as an energy source to drive N2 fixation.

## INTRODUCTION

Deep-sea cold seeps are chemosynthetic ecosystems where fluid seepages are rich in hydrogen sulfide, methane, and other hydrocarbons, providing a source of energy for a diverse range of microbial and benthic communities (1). To date, at least 123 cold seep sites have been documented on continental margins worldwide (2, 3). However, hydrocarbon seepage emits little nitrogen in these carbon-dominated systems, limiting biological productivity and the degradation of non-nitrogenous organic matter (4). Anaerobic methane-oxidizing archaea and sulfate-reducing bacteria consortia are recognized as dominant diazotrophs in cold seep sediments that convert dinitrogen (N_2_) to ammonia for assimilation (5). N_2_ fixation is an ATP-intensive process (6); thus, deep-sea diazotroph may rely on other catabolic pathways for energy, such as the use of oxygen, iron, sulfur, and carbon as terminal electron acceptors (7, 8), indicating that deep-sea N_2_ fixation may be coupled to multiple biogeochemical cycles. For example, sulfate reduction is an important catabolic process fueling diazotrophy in cold seep sediments, which are rich in various organic and inorganic sulfur-containing compounds (8). However, based on studies on N_2_ fixation and sulfur cycling in terrestrial (9) and intertidal zones (10), the pathway of N_2_ fixation driven by sulfur metabolism may change from sulfur reduction to sulfur oxidation when sulfur-containing compounds are released from anoxic sediments into oxygenated overlying seawater through fluid seepage (11).

Sulfur is an essential component of cell. Microorganisms can obtain energy and sulfur source by transforming organic or inorganic sulfur (12, 13) and preferentially utilize organic sulfur over organic carbon in marine systems (14, 15). Moreover, the biomineralization of dissolved organic sulfur (DOS) exerts an important influence on nitrogen, carbon, and phosphorus cycling (12, 16); for example, sulfide-driven denitrification accounts for 36% of the nitrogen loss in the Chilean oxygen minimum zone (17). It is estimated that 1,320 Tg of sulfur is fixed into organic matter by phytoplankton annually, of which ~600 Tg is subsequently released into seawater as DOS through various biological processes (18). In addition, a recent study has shown that a considerable amount of active sulfur-containing organic matter within seep sediments (up to 44.8% of formulas containing sulfur) may be released into the overlying seawater (11). Previous studies have suggested that diazotrophs benefit from *in situ* dissolved organic matter (DOM) pools (19, 20). Indeed, studies have focused on the effects of dissolved organic carbon (DOC), dissolved organic nitrogen (DON), and dissolved organic phosphorus (DOP) on the N_2_ fixation process (21, 22). However, the N_2_ fixation rates and diazotroph community response to sulfur-containing organic matter, as well as how sulfur metabolism drives N_2_ fixation in water column above cold seeps, are unclear.

The Haima cold seep, located on the northwestern slope of the South China Sea, remains active and has diverse biogeochemical cycles, characterized by low oxygen and high DOS (23, 24). At present, previous studies on N_2_ fixation in cold seeps focus on sediments (5, 8). However, the N_2_ fixation in cold seep water column has not yet been clarified. In this study, the diazotroph community structure and N_2_ fixation rate in cold seep water column were determined by *nifH* gene amplicon sequencing techniques and ^15^N_2_ bubble method. Moreover, we conducted *in situ* enrichment experiments to unveiled not just the influence of DOC, DON, and DOP but also the previously neglected effect of DOS on N_2_ fixation. The potential coupling mechanism between N_2_ fixation and sulfur metabolism in cold seep water column was further analyzed via metagenomic analysis. Considering that sulfur is an important electron donor and fluid seepage releases large amounts of sulfur-containing compounds, we hypothesized that specific microbial communities in cold seep water column drive the coupling of N_2_ fixation and sulfur metabolism, with sulfur oxidation potentially serving as the pivotal metabolic process fueling N_2_ fixation in sulfide-rich cold seeps. In addition to cold seeps, environments such as marine hydrothermal vents (25) and coastal wetlands (26) are also rich in sulfur compounds and hydrocarbons, including methane. Our findings will contribute to a better understanding of diazotroph community structure and its associated biogeochemical cycles in these methane- and sulfur compound-rich environments.

## MATERIALS AND METHODS

### Field observation and environmental parameters

**Fig 1:**
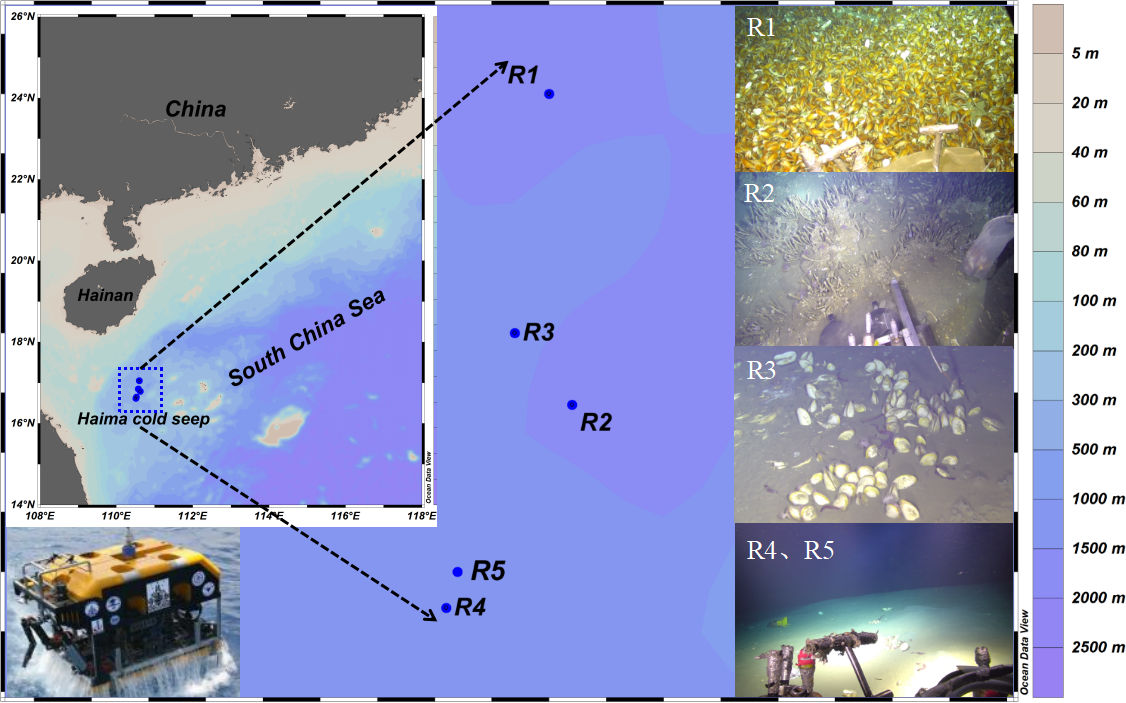
Location and seabed images of different sites of the Haima cold seep, South China Sea. The bottom left corner is an image of the “Haima” remotely operated vehicle (ROV). The right is *in situ* photographs showing typical epibenthic macrofaunal community at each station in the Haima cold seep. R1: mussel bed; R2: large tubeworm; R3: clam bed; R4 and R5 did not exhibit any of the aforementioned benthos. The map of sampling stations was displayed by Ocean Data View version 5.3.0.

Samples were collected in May 2021 from the Haima cold seep using 12 L Niskin bottles assembled on a rosette with conductivity-temperature-depth (CTD) sensor (Sea-Bird 911 plus; Sea-Bird Electronics, Bellevue, WA, USA) at five sites (R1–R5) at a depth of approximately 1,450 m (Fig. 1). Seawater samples were collected at depths of 0, 25, 50, 85, 100, 150/200, 400, 600, 800, 1,000, 1,200, and 1,300–1,400 m. The “Haima” remotely operated vehicle (ROV) was used to collect seawater samples from the bottom layer (1,300–1,400 m). *In situ* temperature, methane (CH_4_) concentration, and dissolved oxygen (DO) concentration were measured using Methane Sensor (METS Methane Sensor; FRANATECH, Germany) and SBE dissolved oxygen sensor (model 43) mounted with the CTD. According to the photos and *in situ* videos taken by camera deployed on the “Haima” ROV in the field, continuous gas bubbles emitted from the sea floor could be clearly observed at R1 and R2, whereas no visible bubbles were recorded at R3, R4, and R5 during this cruise. R1 and R2 are mussel- and tubeworm-dominated seep sites, respectively. R3 is a clam-dominated seep site, whereas R4 and R5 have no macrofaunal community (Fig. 1).

R1 and R2 were classified as active regions, characterized by continuous gas bubbles emitted from the seabed and a CH_4_ concentration near the bottom water of approximately 80 nmol L^−1^. By contrast, R3, R4, and R5 were classified as inactive regions with no gas bubbles and CH_4_ concentration near the bottom water close to 0 nmol L^−1^. Samples collected from the upper layer of each station (150 or 200 m) were denoted as R1-U, R2-U, R3-U, R4-U, and R5-U, and those from the bottom layer (1,300–1,400 m) were denoted as R1-B, R2-B, R3-B, R4-B, and R5-B. In addition, the results from the metagenomes of R1 and R2 was averaged and denoted as R12, representing the results of the active region. R12-U and R12-B were defined as the upper and bottom layers, respectively. Similarly, the results from the metagenomes of R3, R4, and R5 were denoted as R345, representing the results of the inactive region. The methods for nutrient measurements, three-dimensional fluorescence measurements, and the calculation of N deficit (N*) index were described in the supplemental material.

### N_2_ fixation rate measurements

N_2_ fixation rates were determined using the ^15^N_2_ bubble method (27). All polycarbonate bottles were acid-washed and rinsed with Milli-Q water. For the *in situ* N_2_ fixation rates, 2.4 L of bubble-free seawater was decanted into duplicate polycarbonate bottles with septum caps and filled to avoid air contamination. Then, 2.4 mL ^15^N_2_ (abundance: 99 atoms%, chemical purity ≥ 98.5%; Aladdin) was added to each bottle and the incubation bottles were inverted 50 times. Owing to incomplete isotopic gas equilibration (6, 28) and the potential contamination of ^15^N_2_ gas (29), the bubble method may influence N_2_ fixation rate determination. Therefore, long incubation time (24 h) and high purity ^15^N_2_ gas were adopted to minimize this discrepancy in our study (20, 30). Euphotic zone depths were estimated using a Secchi disk, and then irradiances at different depths were calculated. The bottles were placed under the corresponding light intensity by covering them with neutral-density screens and incubated at the same ambient temperature as the sampling depth for 24 h. The initial ^15^N abundance in the particulate organic matter before incubation was recorded by filtering 4 L of seawater through GF/F filters (0.7 µm/25 mm; Whatman). All samples were filtered onto combusted GF/F filters (at 450°C for 4 h) after 24 h of incubation, and the filters were stored frozen (–20°C) for further analysis. In the laboratory, the filters were dried at 60°C for 24 h. The ^15^N abundance was determined using an Elemental Analyzer-Isotope Ratio Mass Spectrometer (Thermo Scientific) at the South China Sea Institute of Oceanography (31). The analytical precision of the δ^15^N was ±0.2‰. N_2_ fixation rates were calculated as follows (27):


$$N_{2}\;fixation\;rates\;\left(nmol\;L^{-1}\;d^{-1}\right)=\frac{1}{\Delta t}\times \;\frac{A_{PON_{f}}-A_{PN_{0}}}{A_{N_{2}}-A_{PON_{0}}}\times \;\frac{PON_{0}\;+\;PON_{f}\;}{2}\;$$

where *t* is the incubation time; *PON_0_* and *PON_f_* are the concentrations of particulate organic nitrogen at the start and end of incubation, respectively; and ${\text{A}}_{{\text{PON}}_{\text{0}}}$, $A_{{PON}_{f}}$, and $A_{N_{2}}$ are the absolute abundances of ^15^N in *PON_0_*, *PON_f_*, and N_2_ pools, respectively. $A_{N_{2}}$ is the isotope ratio of substrate N_2_ during the incubation. In this study, $A_{N_{2}}$ is a theoretical value and was calculated using the amount of added tracer, salinity, and temperature (32), assuming complete dissolution.

### *In situ* enrichment experiments

To explore the effects of DOC, DON, DOP, and DOS on N_2_ fixation rates and diazotroph community composition in the water column above cold seeps, *in situ* enrichment experiments were performed in the upper layers of 150 m at R1 and 200 m at R2 and R5, respectively, as well as in the bottom layer near the seabed at a depth of 1,430 m at R1. Bubble-free seawater from the Niskin bottles was collected in duplicate 2.4 L polycarbonate bottles with septum caps and filled to avoid air contamination. Each treatment group had two replicate samples. Incubation bottles were flushed with helium and filled with seawater by tubing to the bottom of the bottles to reduce oxygen contamination (33, 34). Glucose, urea, and ATP are ubiquitously present in the marine environment and usually served as a representative of DOC, DON, and DOP for investigating the influences of diverse organic compounds on diazotroph or other microorganisms (21, 22, 35–38). For the DOS, previous study indicated that oil seepage may occur at the Haima cold seeps (39), and dibenzothiophene is a model compound of sulfur-containing organic matter in petroleum (40). DOM with different compositions was added to samples from the upper layer at each site as follows: CK (no DOM addition), DON (urea, 48 µM), DOP (ATP, 1 µM), and DOS (dibenzothiophene, 30 µM). In addition, DOC (glucose, 200 µM) was added to the bottom water layer from R1 for incubation. Samples from the upper layers were incubated at 16°C with screens to shade light and at 4°C in darkness. After 48 h of incubation, samples were collected for DNA extraction and N_2_ fixation rate measurements. For N_2_ fixation rate measurements, bubble-free subsamples were decanted into duplicate 1.2 L polycarbonate bottles and 1.2 mL ^15^N_2_ gas was added to each bottle and cultured for 24 h. The methods of DNA extraction, *nifH* gene amplicon sequencing, and analysis were showed in supplementary methods.

### Metagenomic *de novo* assembly, gene prediction, and annotation

Metagenomic shotgun sequencing libraries were constructed and sequenced at Shanghai Biozeron Biological Technology Co. Ltd. Raw sequence reads were subjected to quality trimming using Trimmomatic (http://www.usadellab.org/cms/index.php?age=trimmomatic) to remove low-quality reads. The taxonomy of clean reads for each sample was determined with Kraken2 (41) using a customized Kraken database. The abundance of taxa was estimated using Bracken software (https://ccb.jhu.edu/software/bracken/). Clean sequence reads were generated into a set of contigs for each sample using MegaHit with “--min-contig-len 500” parameters (42). The open reading frames of the assembled contigs were predicted using Prodigal (v2.6.3) (43), and all open reading frames were generated as a set of unique genes after clustering using CD-HIT at 95% identity and 90% coverage (44). The longest sequence of each cluster was considered as the representative sequence of each gene in the unique-gene set. Salmon software (45) was used to determine the read number for each gene. The unique-gene set was searched against the Kyoto Encyclopedia of Genes and Genome (KEGG) database using BLASTX to identify the proteins to retrieve their function annotations, and KEGG pathway analysis was performed to identify the specific biological functions and pathways associated with the genes predicted for each sample.

### Genome binning

The sequencing depth of each contig was calculated using the functional script “jgi_summarize_bam_contig_depths”, a tool of the MetaBAT2 (v.2.12.1) package (46), based on the sorted BAM files generated by using BWA-MEM (v.0.7.17; http://biobwa.sourceforge.net/) and SAMtools (v1.546; http://www.htslib.org/). MetaBAT2 was applied to bin the assemblies with contig depth results under the default parameters (minimum contig length ≥ 1500 bp). CheckM v.1.0.3 (https://ecogenomics.github.io/CheckM/) with the lineage_wf workflow was used to estimate the completeness and contamination of MAGs (47). Dereplication based on the average nucleotide identity >95% was performed using dRep (v2.3.2; parameter: -pa 0.95 -sa 0.99) (48). The “classify_wf” function of the GTDB Toolkit (GTDB-Tk, version r214; https://gtdb.ecogenomic.org/) was introduced to obtain taxonomic information for each MAG. The amino acid sequences encoded by each MAG were also functionally annotated through comparison against the KEGG database.

### Data analysis

The map of sampling stations was displayed by Ocean Data View version 5.3.0 (49). Alpha diversity (Shannon and Simpson), richness (Chao1), and evenness (Pielou's evenness) indices were calculated using the R package “vegan”, where the higher the Simpson index, the lower the community diversity (50). The Wilcoxon rank sum test was applied to determine statistical significance between the control and DOM enrichment treatments. The OTUs with average relative abundance >0.01% across the CK, DON, DOP, and DOS treatment samples were used for network construction (supplemental material). The relationships between the abundance of genes related to nitrogen and sulfur cycling were analyzed using the Spearman correlation with the “ggpmisc” package in R.

## RESULTS

### Environmental parameters

The CH_4_ concentrations at both R1 and R2 increased with depth and reached a maximum near the seabed (R1: 83 nmol L^−1^; R2: 93 nmol L^−1^), but exhibited minimal variation and maintained close to zero throughout the water columns of R3, R4, and R5 (Fig. S1a). CH_4_ concentrations measured by the sensor were lower than those determined using the standard headspace equilibration method (in bottom waters at R1: 1,198.17 nmol L^−1^, R2: 1,680.75 nmol L^−1^, R3: 631.66 nmol L^−1^, R4: 279.00 nmol L^−1^, R5: 281.88 nmol L^−1^) during the same cruise (51). Nevertheless, the measurements by both methods showed the same trend, indicating higher concentrations of CH_4_ at R1 and R2 than at other sites (R3, R4, and R5). The lowest DO was approximately 2.48 mg L^−1^ at depths of 100–200 m and the second minimum was observed at 700–800 m depth at each site (Fig. S1b). The temperature decreased with depth at all sites (Fig. S1c). For *in situ* enrichment experiments, DO was approximately 3.09, 3.740, 3.04, and 2.830 mg L^−1^; CH_4_ concentrations were approximately 12.00, 17.00, 7.00, and 89.00 nmol L^−1^; and temperature was approximately 16.00, 14.00, 15.00, and 3.50°C at R1-U, R2-U, R5-U, and R1-B, respectively.

DON and DOP concentrations generally decreased with depth (Fig. S1d and e). CDOM was high in the euphotic layer and decreased with depth but showed an upward trend near the seabed (Table S1; Fig. S1f through i). DIN and SRP concentrations increased with increasing depth (Fig. S1j and k). The DIN/SRP ratio decreased with depth, from greater than 16 to less than 16 at approximately 100–200 m (Fig. S1i). Overall, the N* also decreased with depth, ranging from a positive value (1–4 µM) at the surface to a negative value (–0.41 to –32.72 µM) below 85 m depth, but increased near the bottom at R1, R2, R4, and R5 (Fig. S1m).

### N_2_ fixation rates and diazotroph community structure

**Fig 2:**
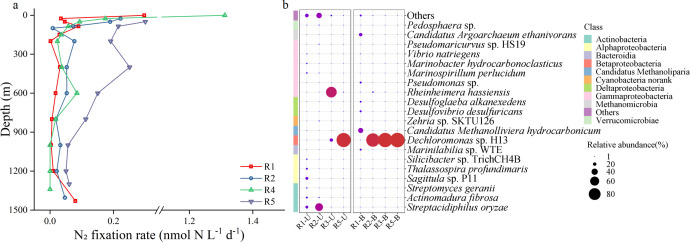
Vertical profile of the (a) N_2_ fixation rates and (b) main diazotrophs at the species level (Top20) in the water column above the Haima cold seep. Color bars indicate class-level taxonomy.

The highest N_2_ fixation rates at each site were all observed in the euphotic layer, reaching 1.31 nmol N L^−1^ d^−1^ at R4 (Fig. 2a). Notably, at R1, N_2_ fixation rates markedly increased from 0.01 nmol N L^−1^ d^−1^ (at 1,200 m depth) to 0.08 nmol N L^−1^ d^−1^ in the bottom waters but showed little change in the inactive region (R3, R4, and R5) (Fig. 2a). A total of 875,332 raw reads were obtained by *nifH* gene high-throughput sequencing, 369,326 sequences were retained after diverse quality control checks, and with 46,165 sequences were retrieved on average for each sample. A total of 535 OTUs were obtained at a 97% sequence similarity level. The sequence percentage of each sample was above 90%, and the average sequence length was between 321 bp. At the species level, *Streptacidiphilus oryzae* dominated the diazotroph community in R1-U and R2-U, with relative abundances of 16.20% and 51.13%, respectively. In R3-U and R5-U, *Rheinheimera hassiensis* and *Dechloromonas* sp. H13 were the dominant diazotrophs, with relative abundances of 72.27% and 96.76%, respectively. In R1-B, the diazotroph community was more diverse and dominated by archaea *Candidatus Methanolliviera hydrocarbonicum* (31.46%) and *Candidatus Argoarchaeum ethanivorans* (19.97%), as well as the sulfate-reducing bacteria *Desulfovibrio desulfuricans* (10.05%). However, the relative abundance of *Dechloromonas* sp. H13 was >90% in the bottom layer at the other sites (Fig. 2b). In the upper layer, the active region (R1 and R2) showed greater alpha diversity, richness, and evenness than those of the inactive region (R3 and R5) (Fig. S2). In the bottom layer, the Shannon index (5.46), Chao1 index (178.50), and Pielou's evenness index (0.73) were the highest in R1-B, whereas the index values of the other stations were comparable (Fig. S2).

### Influence of different DOM composition on N_2_ fixation

**Fig 3:**
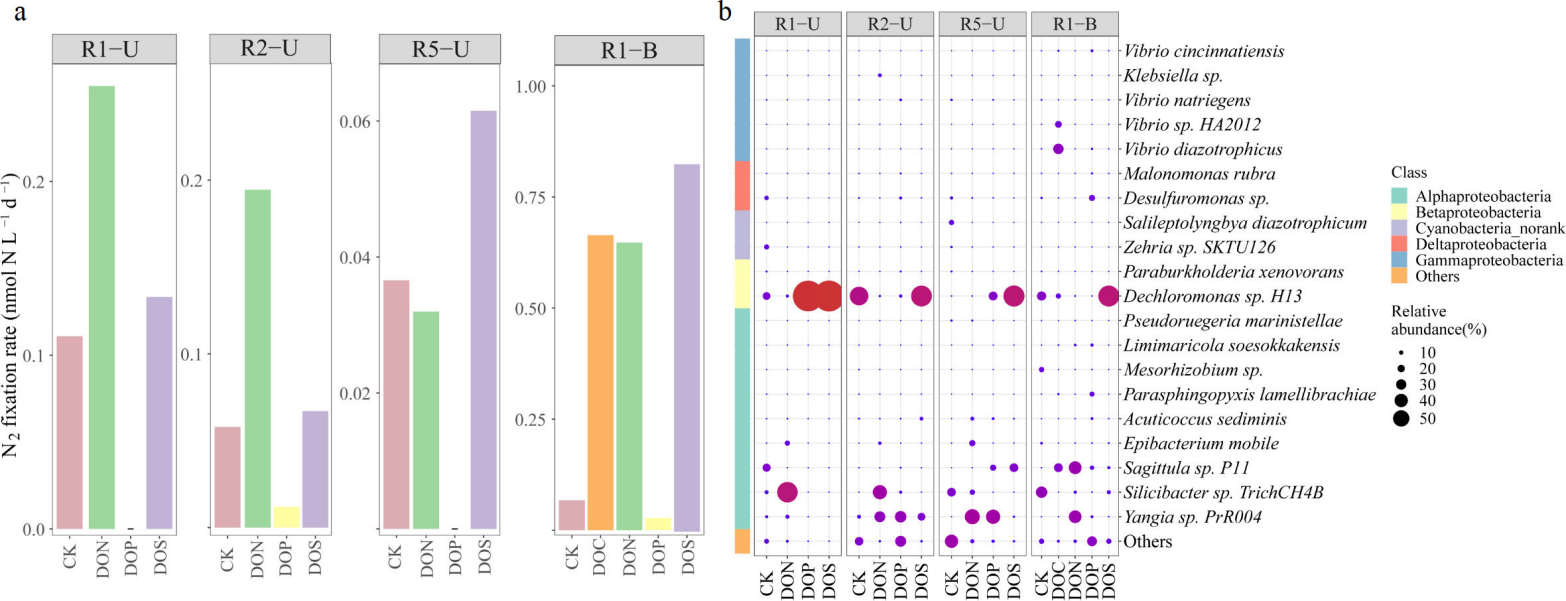
Response of (a) N_2_ fixation rates and (b) main diazotrophs (Top20) at the species level to the addition of different dissolved organic matter (DOM) compositions.

The N_2_ fixation rates of CK in R1-U, R2-U, R5-U, and R1-B were 0.11, 0.06, 0.04, and 0.07 nmol N L^−1^ d^−1^, respectively (Fig. 3a). All DOS treatments increased N_2_ fixation rates (1.20–12.70-fold compared to CK), with the highest rate observed in R1-B (0.86 nmol N L^−1^ d^−1^). DON treatments increased N_2_ fixation rates in R1-U (2.29-fold compared to CK), R2-U (3.36-fold), and R1-B (9.61-fold) but decreased the rate in R5-U (0.87-fold). However, all DOP treatments resulted in lower N_2_ fixation rates (0.42-fold compared to CK) than those of CK treatments (Fig. 3a). In addition, the relative abundances of *Dechloromonas* sp. H13 increased in all DOS treatments (2.08–68.22-fold compared to CK), increasing from 21.31% (CK) to 98.80% in R1-U, 57.58% (CK) to 65.18% in R2-U, 0.97% (CK) to 65.94% in R5-U, and 26.22% (CK) to 65.55% in R1-B (Fig. 3b). DON treatments increased the relative abundance of *Silicibacter* sp. TrichCH4B (64.11%) in R1-U, *Silicibacter sp*. TrichCH4B (42.83%), and *Yangia* sp. PrR004 (31.99%) in R2-U; *Yangia* sp. PrR004 (45.71%) in R5-U; and *Sagittula* sp. P11 (38.40%) and *Yangia* sp. PrR004 (37.92%) in R1-B. For DOP treatments, *Dechloromonas* sp. H13 (98.11%), *Yangia* sp. PrR004 (34.08%), *Yangia* sp. PrR004 (43.08%), and *Desulfuromonas* spp. (15.79%) were the dominant diazotrophs in R1-U, R2-U, R5-U, and R1-B, respectively (Fig. 3b). DOC treatments increased the relative abundance of *Vibrio sp*. HA2012 (30.33%), *Vibrio diazotrophicus* (18.27%), and *Sagittula sp. P11* (25.05%) in R1-B (Fig. 3b). Overall, the average Shannon index and Pielou's evenness index in the DON (3.91 and 0.50) and DOP (3.86 and 0.50) treatments were basically equal to those in the CK treatments (3.93 and 0.53), whereas the DOS (2.12 and 0.29) treatment values were significantly lower (Wilcoxon test, Shannon: *P* < 0.01, Pielou's evenness: *P* < 0.01) than those of CK treatments (Fig. S3). The trend of the Simpson index was opposite to Shannon and Pielou's evenness indices, with that of DOS treatments (0.46) being significantly higher than the CK treatments Simpson index (0.18; Wilcoxon test, *P* < 0.01).

The co-occurrence network showed that nodes within the top five modules accounted for 66.55% (CK), 72.17% (DON), 72.91% (DOP), and 91.20% (DOS) of the total nodes. In the DON, DOP, and DOS treatments, the nodes belonging to orders Rhodobacterales, Vibrionales, and Rhodocyclales in five main modules were increased, suggesting these groups as key linker to maintain network stability. The topological properties of the DOS and DON networks decreased compared to those of the CK network (nodes: 296, total edges: 1771), but increased in the DOP network (Fig. S4; Table S2). The Zi-Pi plots showed that most OTUs in the CK, DON, DOP, and DOS networks were peripheral, with only a few OTUs serving as connectors and module hubs and no OTUs acting as network hubs (Fig. S5a). Both the DOP and DOS networks had one module hub represented by OTU105 (*Limimaricola soesokkakensis*, affiliated with Rhodobacterales) and OTU238 (*Dechloromonas* sp. H13, affiliated with Rhodocyclales), respectively (Fig. S5b). Notably, *Dechloromonas* sp. H13 was numerically dominant and played a key role in the ecological network after the addition of DOS.

### Metagenomic analysis of nitrogen and sulfur metabolisms

**Fig 4:**
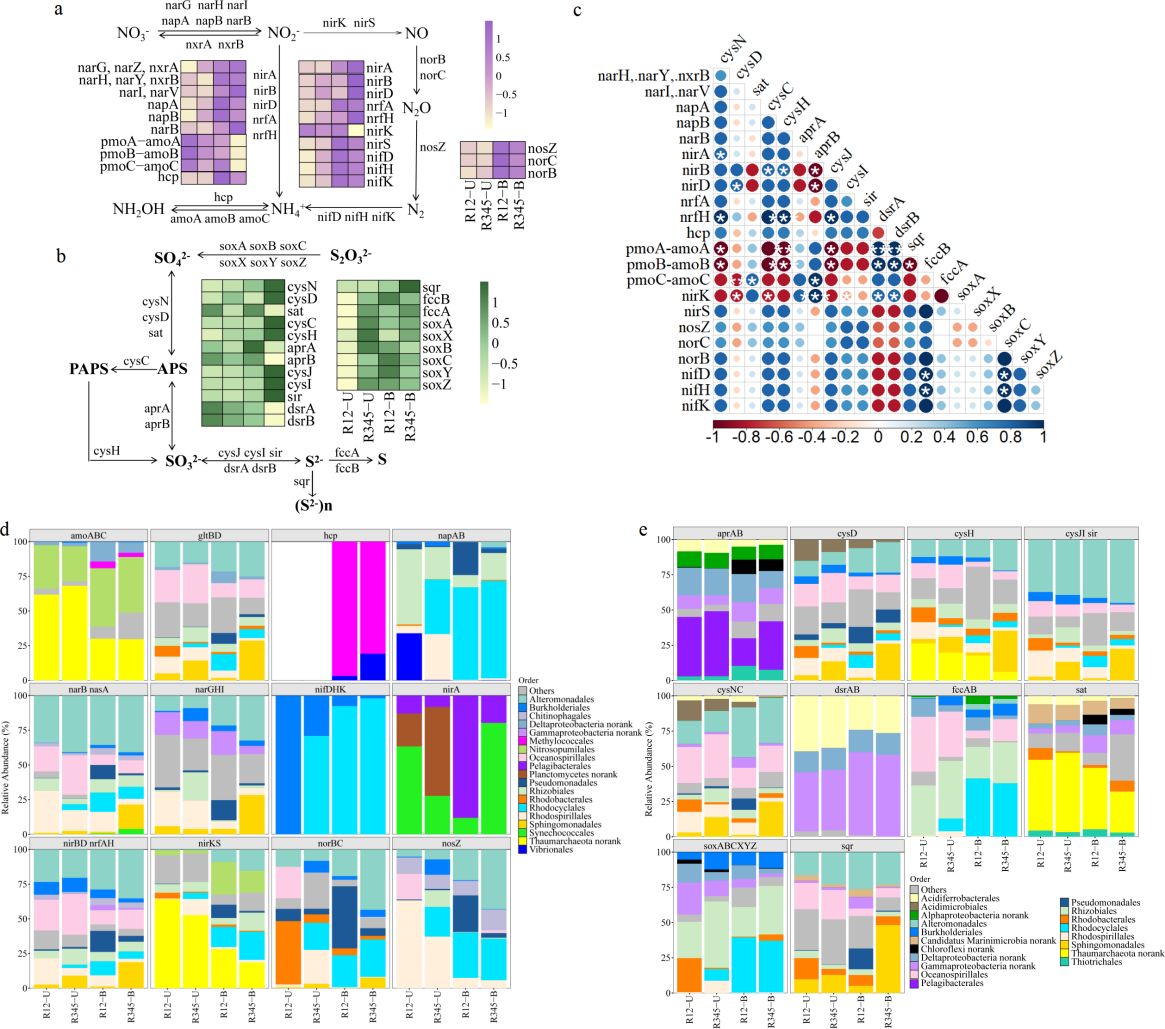
Nitrogen and sulfur metabolisms in the water column above the Haima cold seep. Row-scaled abundance of genes involved in (a) nitrogen and (b) sulfur cycling in all samples. (c) Spearman's correlation of genes involved in nitrogen and sulfur cycling. Each dot represents a correlation between two metabolisms with red and blue representing negative and positive correlations, respectively. Microbial taxa of key genes involved in (d) nitrogen and (e) sulfur cycling and their relative abundance in each sample. “**” means *P* < 0.01, “*” means *P* < 0.05.

In general, nitrogen and sulfur cycling genes were more abundant in the bottom layer than upper layer (Table S3; Fig. 4a and b). Among these, genes involved in denitrification (*nirKS*, *NorBC*, and *nosZ*), nitrification (*amoABC* and *hcp*), N_2_ fixation (*nifDHK*), dissimilatory sulfate reduction and oxidation (*aprAB*, *dsrAB*, *sat*, *met3*, and *APA1_2*), and SOX system (*soxABCXYZ*) were more abundant in the bottom layer of the active (R12-B) than in the non-active region (R345-B). Conversely, genes associated with assimilation (*narB*, *nasA*, and *nirA*) and dissimilation (*narGHI*, *napAB*, *nirBD*, and *nrfAH*) related to nitrate reduction, assimilatory sulfate reduction (*cysCDHNIJ*), and sulfide cycling (*fccAB* and *sqr*) exhibited higher abundance in the bottom layer of the non-active region (Fig. 4a and b). Furthermore, a strong correlation was observed between nitrogen and sulfur metabolisms (Fig. 4c). For example, the *nifDHK* genes involved in N_2_ fixation were significantly positively correlated with *fccB* and *soxC* genes involved in sulfur compound oxidation (Spearman correlation, r = 1.00, *P* < 0.05) (Fig. 4c). Notably, Rhodocyclales (mostly classified closest to *Dechloromonas* clades) was highlighted as an important group that harbors genes for both nitrogen (N_2_ fixation and denitrification) and sulfur metabolisms (sulfide oxidation and SOX system) in the bottom layers (Fig. 4d and e).

### Genomic analysis of *Dechloromonas* sp

**Fig 5:**
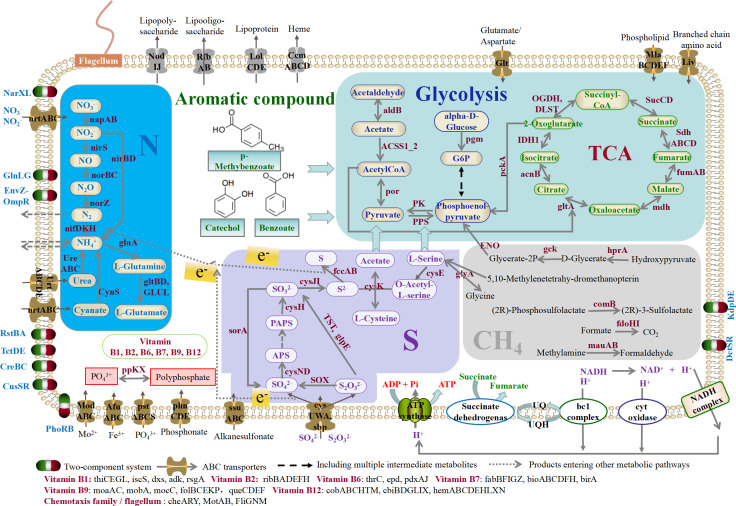
Genomic analysis of *Dechloromonas* sp. Predicted metabolic pathways and properties of *Dechloromonas* sp. Major pathways for carbohydrate, nitrogen, and sulfur metabolisms, along with genes related to chemotaxis, vitamin biosynthesis, ABC transporters, and two-component systems are shown.

A MAG of *Dechloromonas* sp. was obtained by metagenome assembly and binning with 97.95% completeness and 0% contamination. The genome of this strain was highly similar to that of *Dechloromonas* sp. CZR5 (average nucleotide identity = 94.50%) (Fig. S6a). The estimated genome size was 4.37 Mbp, with 81 contigs and 4,243 genes. The calculated G + C content of chromosomal DNA was 62.36% (Fig. S6b). A total of 75 tRNA genes were found in the genome (Table S4). Based on the KEGG annotation (Table S5), the genome contained common carbohydrate metabolic pathways, such as the citrate cycle (TCA cycle) and glycolysis (Fig. 5). Furthermore, it contained genes related to nitrogen metabolism, including complete nitrogenase gene clusters (*nifDHK*, *fixAB*, and *rnfABCDEG*), denitrification (*nirS*, *norBC*, and *nosZ*), and dissimilatory nitrate reduction (*napAB* and *nirBD*) (Fig. 5). The genome also included genes associated with sulfur metabolism, such as assimilatory sulfate reduction (*cysCDHNIJ*) and sulfite/thiosulfate oxidation system (*soxABCXYZ*) (Fig. 5). The genome contained a variety of ATP-binding cassette (ABC) transporters, such as *UrtABCDE* and *CysPUWA*, which are involved in membrane transportation of urea and SO_4_^2−^/S_2_O_3_^2−^, respectively. Two-component systems involved in signal transduction that enable bacteria to respond to changes in their habitat or intracellular conditions were identified in the genome; these include *PhoR−PhoB*, a phosphate regulon sensor; *NarX−NarL* and *GlnL−GlnG*, nitrate/nitrite regulation sensors; and *EnvZ−OmpR*, osmotic stress sensors (Fig. 5). Genes involved in the metabolism of aromatic compounds, such as the *ben* and *dmp* gene families that catalyze benzoate to pyruvate, were also identified in the genome (Fig. 5).

## DISCUSSION

### Active N_2_ fixation in the water column above the Haima cold seep

N_2_ fixation is an important process that can compensate for nitrogen loss resulting from denitrification and anammox (52, 53). The consortium of anaerobic methane-oxidizing archaea and sulfate-reducing bacteria is recognized as a pivotal diazotrophic system that crucially contributes new nitrogen to hypoxic, organic matter-rich cold seep sediments (5, 54). N_2_ fixation rates reached 26.6 nmol N_2_ g_dw_^−1^ in the active CH_4_ seepage sediments of Costa Rica (5). Nevertheless, the diazotrophic community structure and N_2_ fixation rates in the water column above cold seeps have not been determined. The present study revealed N_2_ fixation rates of up to 1.31 nmol N L^−1^ d^−1^ in surface waters in the Haima cold seep, which was similar to the maximum N_2_ fixation rate (1.28 nmol N L^−1^ d^−1^) within upper 200 m in non-cold seep region of the South China Sea (55), but lower than that observed in the oxygen-deficient zone of the Eastern Tropical South Pacific (56–58). In cold seeps, most of the oxygen in bottom waters is consumed by the oxidation of reduced chemical compounds and biological respiration (59, 60). Diazotrophs, such as *Dechloromonas* and *Vibrio* species in aggregate (61) and ANME-2 and sulfate-reducing bacteria (SRB) *Desulfosarcina*/*Desulfococcus* consortia in deep-sea sediments (54), benefit from low O_2_ concentrations because nitrogenase is an oxygen-sensitive protein (62, 63). In the present study, we found that SRB *Desulfovibrio* and *Dechloromonas* were also distributed in the bottom waters (Fig. 2b). This distribution may be related to the release of suspended organic particles from sediments into the overlying waters (64) and the attachment of these diazotrophs to organic-rich aggregates (65). It is easier to form a lower O_2_ concentration or even anoxic microenvironment on organic particles with continuous high-intensity CH_4_ seepage, which is conducive to biological N_2_ fixation in oxygenated waters (66, 67). In this study, analysis of the *nifH* gene revealed that *Dechloromonas* sp. H13 was the most abundant diazotroph in the water column above the cold seep (Fig. 2b), especially in the bottom layers of R2, R3, and R5 (relative abundance greater than 90%), which resulted in significantly lower alpha diversity in R2-B, R3-B, and R5-B than in R1-B (Fig. S2). The genus *Dechloromonas* has been identified in free-living or particle-associated forms in various environments, such as low-oxygen waters (68, 69), estuarine sediments (70), and eutrophic rivers (71). Members of the genus *Dechloromonas* are well-known aromatic compound-degrading and facultative anaerobic nitrate-reducing bacteria (72, 73); however, further studies are necessary to confirm the diazotrophic function of *Dechloromonas* and evaluate its ecological role and biogeochemical significance in marine environments.

### Diazotrophic responses to DOM additions

Heterotrophic diazotrophs rely on external DOM as their energy source (74). The composition of DOM varies among different sources with specific non-metallic elements such as nitrogen, sulfur, and phosphorous. In this study, an on-site investigation revealed that the increase in N_2_ fixation rates coincided with high CDOM concentrations at depths below 1,200 m (Fig. 2a; Fig. S1f through i). Previous studies also have shown that diazotrophs benefit from *in situ* DOM pools and that N_2_ fixation rates are positively correlated with organic matter in deep water (19, 55, 58). Furthermore, DOS significantly increased the N_2_ fixation rates and the relative abundance of *Dechloromonas* sp. H13 (Fig. 3), presumably owing to availability of energy generated by sulfur-based reactions for N_2_ fixation (12). For example, the oxidation of sulfide to sulfate releases 209–701 kJ mol^−1^ (75), which may be utilized for N_2_ fixation according to the thermodynamic model for free energy yield (76). Thus, when sulfur-containing organic matter in sediments continues to be released from sediments into the overlying waters (11), the correlation between N_2_ fixation and the sulfur cycle may be strengthened. DON also increased the N_2_ fixation rates (Fig. 3a), consistent with previous observations in the mesopelagic zone of the Bismarck and Solomon Seas (i.e., a 1–2-fold increase in N_2_ fixation rates with amino acid addition) (19). However, in our study, higher DON concentrations were detected in deep waters (approximately 10 µM below 600 m depth) than in the North Pacific Ocean (2–3 µM below 900 m depth) (77) but were consistent with the trend in a seepage site in the southeast Mediterranean Sea (4–6 µM below 400 m depth) (64), which may be related to the release of nitrogen-containing organic matter from sediments to the water column above the seep (11, 78). Moreover, we found that active N_2_ fixation (Fig. 2a) could occur in dissolved nitrogen-rich waters (Fig. S1j), suggesting that the presence of nitrogen did not always inhibit N_2_ fixation and heterotrophic diazotrophs in the deep sea may benefit from organic matter (79). However, N_2_ fixation rates significantly decreased with all DOP treatments (Fig. 3a), which is in line with findings in the hypoxic zone of the Eastern Tropical South Pacific (21). Based on the DIN/SRP ratio (<16) and negative values of N* (Fig. S1m and j), we speculated that this phenomenon may be related to the depletion of nitrogen through denitrification, resulting in a sufficiency of phosphorus relative to the availability of nitrogen and a potential nitrogen limitation (80, 81). Traditionally, denitrification should be inhibited at these oxygen concentrations (DO: 2–4 mg L^−1^) in this study (82). However, owing to the presence of suspended organic particles in the overlying oxygenated water (4), the occurrence of denitrification under oxic conditions may be linked to particles with a low-oxygen microenvironment according to a model (83), laboratory experiments (84), and environmental data (85). It should be noted, however, that we only tested a limited number of different DOM sources (i.e., DOC, DON, DOP, and DOS). We expect that a larger and more abundant DOM pool in the deep sea and that other DOM sources may have different effects on diazotrophs; therefore, further research is necessary to expand our findings.

### Potential of the genus *Dechloromonas* to couple N_2_ fixation and sulfur cycling

**Fig 6:**
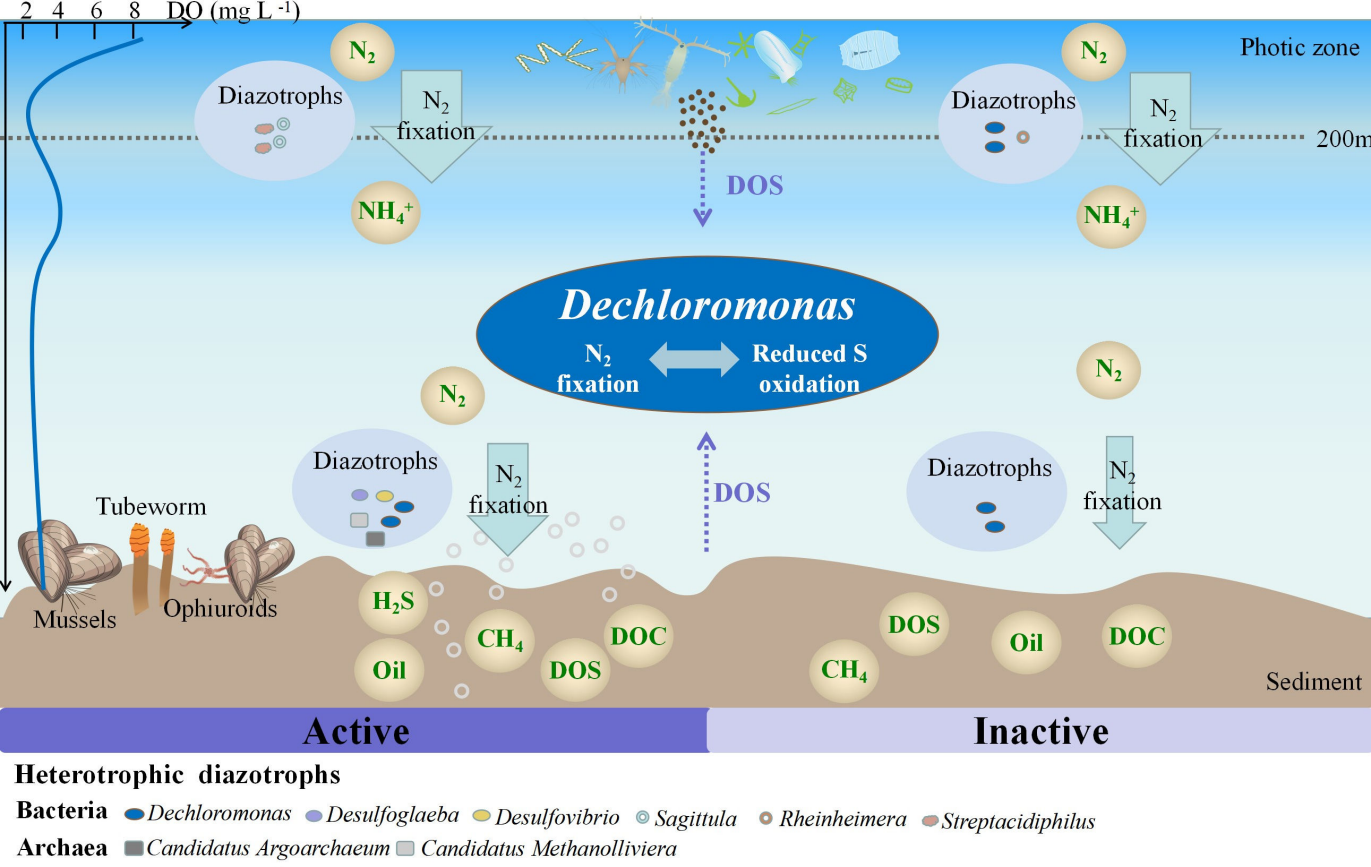
Schematic representation of the N_2_ fixation in water above cold seeps and N_2_ fixation under DOS loading. The bolder the blue arrows, the higher the N_2_ fixation rates. DOS is mainly derived from cold seepage and euphotic plankton production. DO, dissolved oxygen; DOC, dissolved organic carbon; DOS, dissolved organic sulfur.

Previous studies have shown that some members of the *Dechloromonas* possess sulfate reduction pathway and sox system and can uptake sulfur compounds (e.g., SO_4_^2-^) from the surroundings (73, 86). Our study also suggests that *Dechloromonas* has the potential for metabolizing sulfur compounds and may serve as a keystone taxon that plays a crucial role in integrating N_2_ fixation and sulfur cycling. Specifically, our metagenomic analysis indicated a positive correlation between N_2_ fixation (*nifDHK*) and sulfur oxidation genes (*fccB* and *soxC*) (Fig. 4c). These genes were predominantly contributed by Rhodocyclales (OTUs mostly closely affiliated with the *Dechloromonas* clade) in the bottom layer (Fig. 4d and e). The simultaneous increase in N_2_ fixation rates and relative abundance of *Dechloromonas* after the addition of DOS further confirmed the coupling between N_2_ fixation and sulfur cycling. Moreover, the genome of *Dechloromonas* sp. contained genes involved in N_2_ fixation (*nifDHK*), sulfide oxidation (*fccAB*), and sulfite/thiosulfate oxidation (*soxABCXYZ*) (Fig. 5). Hence, we proposed that *Dechloromonas* may serve as a multifunctional coupler in the water column above the Haima cold seep, facilitating the coupling of nitrogen and sulfur cycling (Fig. 6). In addition, based on the *in situ* benthic macrofauna and their endosymbiotic bacteria, we could also infer that *Dechloromonas* has the ability to metabolize sulfur compounds. Previous studies have indicated that fluid seepage is accompanied by the release of abundant hydrogen sulfide from the seafloor to bottom waters (4, 87) and that the distribution of microbes and animal-microbe symbioses can also indirectly reflect, to a certain extent, sulfides (88). Tubeworms at R2 and clams at R3 are symbiotic with sulfur-oxidizing bacteria (89, 90) whereas mussels at R1 mainly coexist with methane-oxidizing bacteria (91, 92), suggesting that the sulfide concentration at R2 may be higher than that at R1. Therefore, the higher relative abundance of *Dechloromonas* at R2 compared to that at R1 may be attributed to its ability to oxidize reduced sulfur-containing compounds.

### Conclusion

In summary, the interactions between different organic matter compositions and N_2_ fixation were investigated in the water column above the Haima cold seep. Active N_2_ fixation occurred *in situ*, and *Dechloromonas* was the most abundant diazotroph based on *nifH* gene sequencing. Furthermore, the response of N_2_ fixation rates and *Dechloromonas* [containing genes involved in N_2_ fixation (*nifDHK*) and sulfur compound oxidation (*fccAB*, *soxABCXYZ*)] to DOS highlighted the potential coupling between N_2_ fixation and sulfur metabolism. In this study, we revealed that the *Dechloromonas* genus plays a crucial role as diazotrophs and acts as a mediator in sulfur-driven nitrogen fixation process in cold seeps water column. These findings may have broad biogeochemical implications in similar environments containing abundant sulfur-containing compounds and hydrocarbons, such as deep-sea hydrothermal vents and wetland systems.

## Data Availability

All sequences of the *in situ nifH* gene and metagenome obtained in this study have been deposited in the National Center for Biotechnology Information (NCBI) under BioProject accession number PRJNA944722.

## SUPPLEMENTAL MATERIAL

Supplemental material spectrum.00536-24-s0001.pdf Fig. S1 to S6; Tables S1 to S5.

ASM does not own the copyrights to Supplemental Material that may be linked to, or accessed through, an article. The authors have granted ASM a non-exclusive, world-wide license to publish the Supplemental Material files. Please contact the corresponding author directly for reuse.
