# Supplementary material for: Cold seep nitrogen fixation and its potential relationship with sulfur cycling
Source: Microbiol Spectr. 2024 Aug 22;12(10):e00536-24. doi: 10.1128/spectrum.00536-24 (PMC11448218; doi:10.1128/spectrum.00536-24)
Supplement: Supplemental material — Fig. S1 to S6; Tables S1 to S5. [file spectrum.00536-24-s0001.pdf]

Cold seep nitrogen fixation and its potential relationship with sulfur cycling

Qiumei Quan[1,2], Jiaxing Liu[1], Xiaomin Xia[1], Si Zhang[1,3], Zhixin Ke[1], Yehui Tan[1,2*]

[1]South China Sea Institute of Oceanology, Chinese Academy of Sciences, Guangzhou 510301, China

[2]University of Chinese Academy of Sciences, Beijing 100049, China

[3]Southern Marine Science and Engineering Guangdong Laboratory (Guangzhou), Guangzhou 510301, China

Qiumei Quan and Jiaxing Liu should be considered joint first authors.

Correspondence

*Yehui Tan: South China Sea Institute of Oceanology, Chinese Academy of Sciences, Guangzhou 510301, China; Email: tanyh@scsio.ac.cn

The supplementary information includes:

- Materials and methods
- 6 figures (Fig. S1 to S6)
- 5 tables (Table S1 to S5)

Fig. S1 Vertical profile of environmental parameters: (a) CH_4, (b) DO, (c) temperature, (d, e) dissolved organic nutrients (DON and DOP), (f–i) CDOM compounds (C1: biological production, C2: PAH-protein-like, C3: tryptophan-like, C4: humic-like), and (j–m) inorganic nutrient (DIN, SRP, DIN/SRP, and N*) at five sites in the water column above the Haima cold seep (R1 and R2 in the active region; R3, R4, and R5 in the inactive region). CH4, methane; DO, dissolved oxygen; DON, dissolved organic nitrogen; DOP, dissolved organic phosphorus; CDOM, chromophoric dissolved organic matter; DIN: Dissolved inorganic nitrogen; SRP: Soluble reactive phosphate; DIN/SRP: ratio of DIN to SRP; N*: N deficit index.

Fig. S2 Mean alpha diversity (Shannon), richness (Chao1) and evenness (Pielou's evenness) indices in the water column above the Haima cold seep.

Fig. S3 Mean alpha diversity (Shannon and Simpson), richness (Chao1) and evenness (Pielou's evenness) indices in CK, DON, DOP, DOS, and DOC treatment. DOC, DON, DOS, and DOP: dissolved organic matter containing carbon, nitrogen, sulfur, and phosphorous, respectively; CK, no addition of dissolved organic matter.

Fig. S4 Co-occurrence networks of the diazotroph community in (a) CK, (b) DON, (c) DOP, and (d) DOS treatments. Each node represents an OTU. OTU with relative abundances of >0.01% were selected. Node size is proportional to the relative abundance. Edges between nodes represent the relationships

among OTUs. Positive links are indicated in red and negative links are indicated in green. The stack diagram shows the diazotroph in the main modules at the order level. DON, DOP, and DOS: dissolved organic matter containing nitrogen, sulfur, and phosphorous, respectively; CK, no addition of dissolved organic matter. Module1-Module5 (M1-M5) represents the five modules with the highest number of nodes in each network. The five modules are displayed in different colors, and other modules are displayed in grey. The percentage represents the number of nodes in each module as a proportion of all nodes.

Fig. S5 Zi-Pi plot showing the distribution of all operational taxonomic units (OTUs) based on their topological properties. (a) Each dot symbol represents an OTU. All dots were distributed among the following four subcategories: peripherals (grey), connectors (red), module hubs (light blue), and network hubs (light purple). (b) Species composition of the connectors at the order level. CK, no DOM addition; DON, dissolved organic nitrogen; DOP, dissolved organic phosphorus; DOS, dissolved organic sulfur.

Fig. S6 Genomic analysis of *Dechloromonas* sp. (a) Average nucleotide identity heatmap between *Dechloromonas* sp. and other strains of the same genus. (b) Genetic map of the genomes of *Dechloromonas* sp.

Table S1. The excitation and emission wavelengths of four fluorescent components in chromophoric dissolved organic matter (CDOM) in the water column above the Haima cold seep.

Table S2. Topological properties of diazotroph community co-occurrence networks based on the *nifH* gene.

Table S3. Distributions of key functional genes (nitrogen and sulfur metabolism) in the metagenomes.

Table S4. tRNAs identified in the genome of *Dechloromonas* sp..

Table S5. Kyoto Encyclopedia of Genes and Genome annotation of a metagenome-assembled genomes belonging to *Dechloromonas* sp.

Materials and methods

Nutrient measurements

Nitrite (NO_2^-), nitrate (NO_3^-), and ammonium (NH_4^+) were analyzed using a flow injection analyzer (QuichChem8500; Lachat Inc., Loveland, CO, USA) following standard colorimetric methods(1). The soluble reactive phosphate (SRP) was measured using a spectrophotometer according to the standard molybdenum blue method (detection limit of 0.05 μmol L^{-1}). Total dissolved nitrogen (TDN) and total dissolved phosphorus (TDP) were digested with a $K_2S_2O_8$ solution at 120 °C for 30 min before measurement using the flow injection analyzer(1). The TDN and TDP standards were processed in parallel with the water samples. Dissolved inorganic nitrogen (DIN) is the sum of NO_2^-, NO_3^-, and NH_4^+ concentrations. The dissolved organic nitrogen (DON) concentration is the difference between TDN and DIN. The dissolved organic phosphorus (DOP) concentration was calculated as the difference between TDP and SRP.

Three-dimensional fluorescence measurements

The excitation–emission matrix fluorescence spectra of the chromophoric dissolved organic matter (CDOM) were measured using a Hitachi F-4600 fluorescence spectrometer (Hitachi High-Technologies, Tokyo, Japan) with a 700-voltage xenon lamp at scanning ranges of 200–450 nm excitation and 225–550 nm emission. Milli-Q water was used as a blank control to test the stability of the instrument. Excitation–emission matrix values were corrected using the MATLAB 10.0 software package (Math Works, Natick, MA, USA). Statistical decomposition was used to extract the most representative fluorescent components from the excitation–emission matrix dataset(2). The CDOM components was determined by previous study(3): biologically produced fresh protein-like (C1)(4), polycyclic aromatic

hydrocarbon mixture (C2)(5), tryptophan-like (C3)(5), and microbial-derived humic-like (C4) (Table S1)(6).

N deficit (N*) index

The fixed inorganic N deficit (N*) has been widely used to evaluate the N budget in the ocean(7, 8). The positive values of N* are interpreted as excess N through nitrogen fixation, whereas negative values of N* indicate a loss of N through denitrification(9, 10). The N* was calculated as follows: $N^* = \left((NO_3 + NO_2) - 16 \times \left(PO_4^{3-}\right)\right) + 2.9\ \mu M.$

DNA extraction, *nifH* gene amplicon sequencing, and analysis

Samples from upper and bottom layers as well as in situ enrichment experiments were extracted using the E.Z.N.A® Water DNA Kit (OMEGA, USA). Nested polymerase chain reaction (PCR) amplification of *nifH* gene was performed using nifH3 and nifH4 primers in the first PCR followed by a second amplification using nifH1 and nifH2 primers(11). Sequencing and annotation were performed at Biozeron Biological Technology Co., Ltd. (Shanghai, China) using the Illumina HiSeq 2500 platform (Illumina, San Diego, CA, USA). Clean sequences were assigned to the same operational taxonomic units (OTUs) defined at 97% similarity using the UCLUST algorithm. Each representative OTU was annotated against the National Center for Biotechnology Information (NCBI) non-redundant (NR) protein database using BLASTX to identify their phylogenetic affiliations.

Molecular ecological network analysis

OTUs displaying an average relative abundance exceeding 0.01% across the CK, DON, DOP, and DOS treatment samples were chosen for network construction. Spearman coefficients (r) between OTUs were calculated the "Hmisc" package in R. Robust ($|r| > 0.60$) and statistically significant (BH-adjusted

P <0.01) correlations were incorporated into the network analyses. Network parameters, including betweenness, edges, degree, closeness, and vector were generated using the “igraph” package in R. Network visualization was performed using Gephi 0.9.2 software with an undirected network, Fruchterman-Reingold layout, and modularity class partition. Zi-Pi plots were used to identify the key species that may play an important role in maintaining the stability of the microbial community structure in the ecological network. All nodes in the network were distributed among the following four subcategories: peripherals (Zi score < 2.5; Pi score < 0.62), connectors (Zi score < 2.5; Pi score > 0.62), module hubs (Zi score > 2.5; Pi score < 0.62), and network hubs (Zi score > 2.5; Pi score > 0.62). Nodes in the latter three subcategories are considered keystone microbial species that play a significant role in the network(12).

Figure

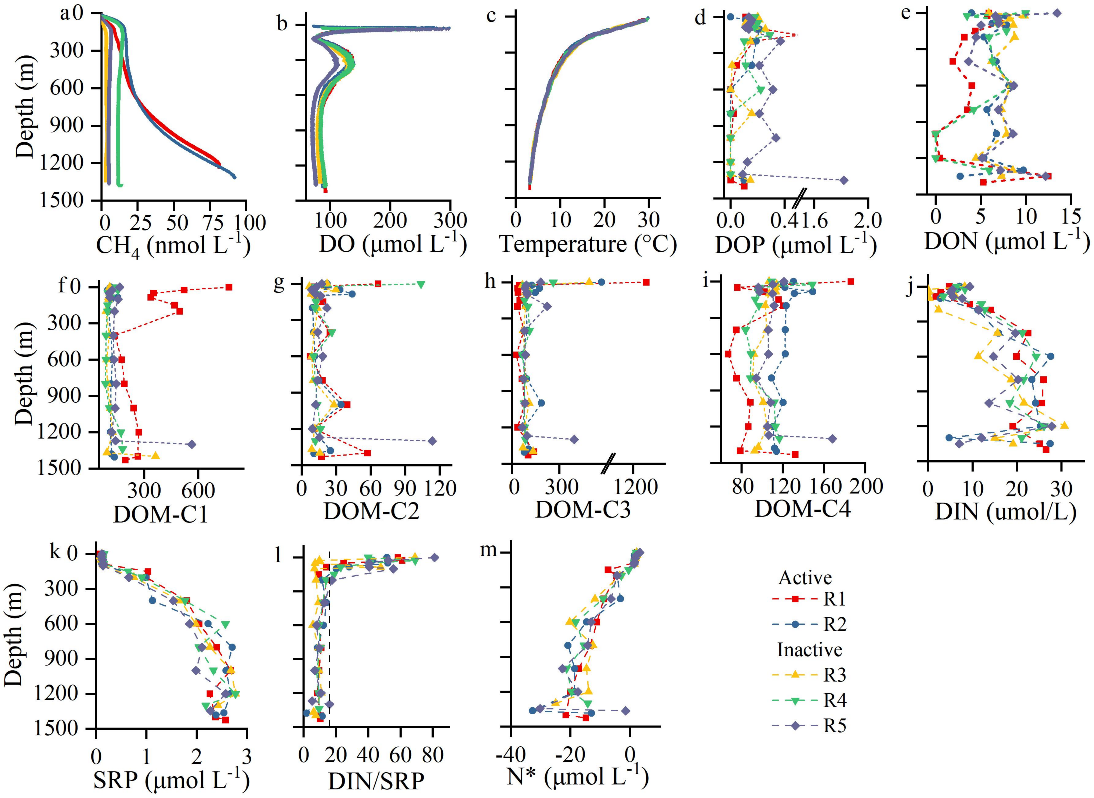

Fig. S1 Vertical profile of environmental parameters: (a) CH_4, (b) DO , (c) temperature, (d, e) dissolved organic nutrients (DON and DOP), (f–i) CDOM compounds (C1: biological production, C2: PAH-protein-like, C3: tryptophan-like, C4: humic-like), and (j–m) inorganic nutrient (DIN, SRP, DIN/SRP, and N*) at five sites in the water column above the Haima cold seep (R1 and R2 in the active region; R3, R4, and R5 in the inactive region). CH_4, methane; DO, dissolved oxygen; DON, dissolved organic nitrogen; DOP, dissolved organic phosphorus; CDOM, chromophoric dissolved organic matter; DIN: Dissolved inorganic nitrogen; SRP: Soluble reactive phosphate; DIN/SRP: ratio of DIN to SRP; N*: N deficit index.

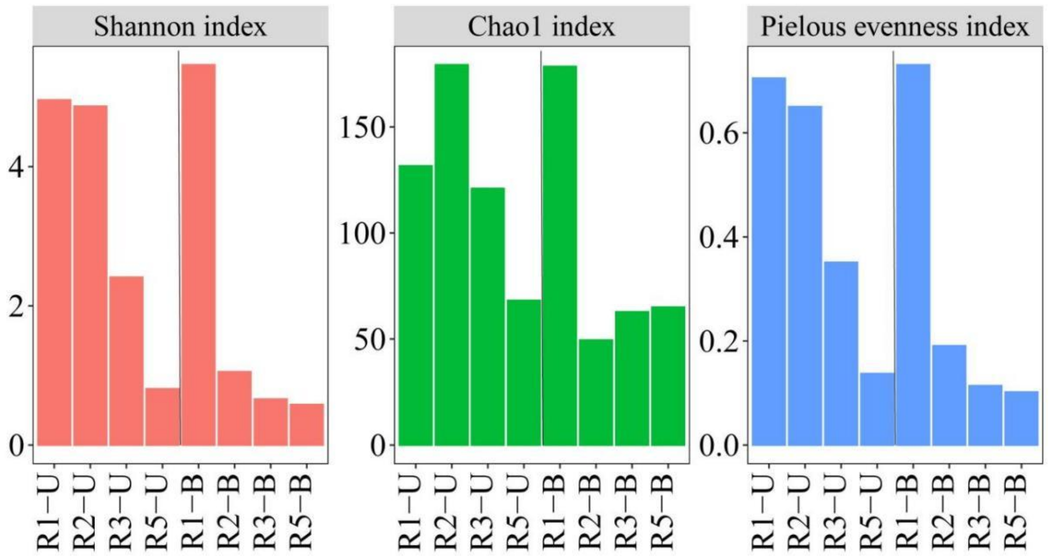

Fig. S2 Mean alpha diversity (Shannon), richness (Chao1) and evenness (Pielou's evenness) indices in the water column above the Haima cold seep.

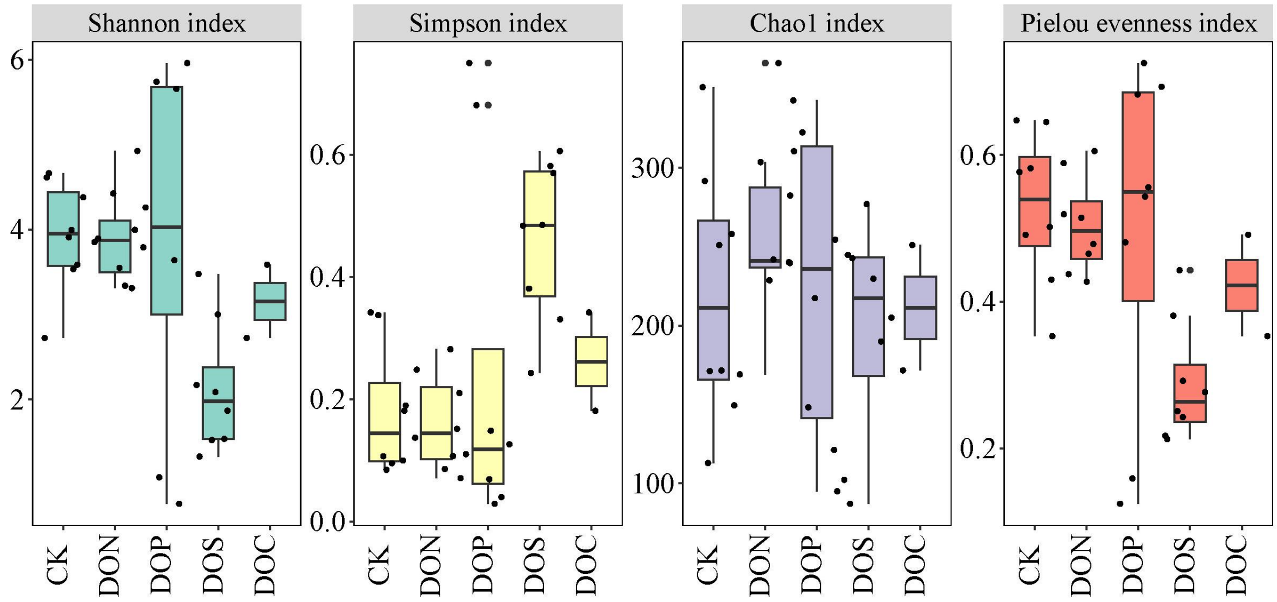

Fig. S3 Mean alpha diversity (Shannon and Simpson), richness (Chao1) and evenness (Pielou's evenness) indices in CK, DON, DOP, DOS, and DOC treatments. DOC, DON, DOS, and DOP: dissolved organic matter containing carbon, nitrogen, sulfur, and phosphorous, respectively; CK, no addition of dissolved organic matter.

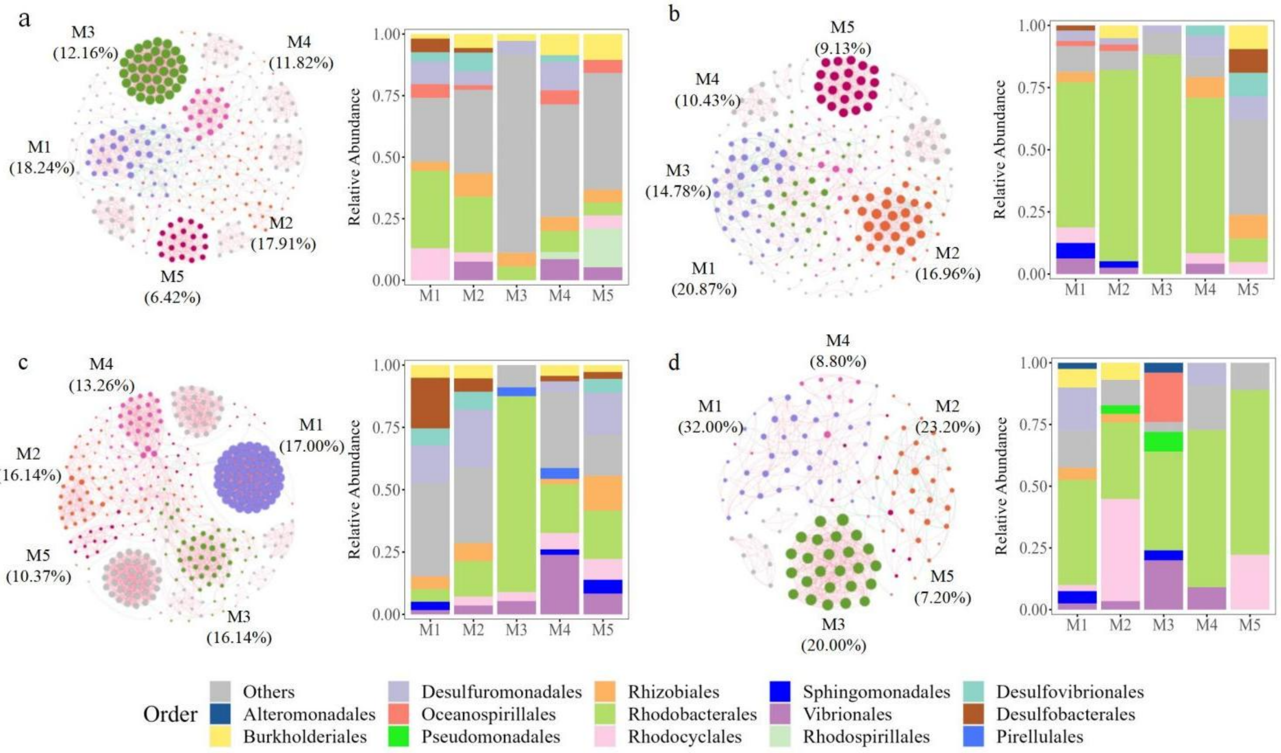

Fig. S4 Co-occurrence networks of the diazotroph community in (a) CK, (b) DON, (c) DOP, and (d) DOS treatments. Each node represents an OTU. OTU with relative abundances of >0.01% were selected. Node size is proportional to the relative abundance. Edges between nodes represent the relationships among OTUs. Positive links are indicated in red and negative links are indicated in green. The stack diagram shows the diazotroph in the main modules at the order level. DON, DOP, and DOS: dissolved organic matter containing nitrogen, phosphorous, and sulfur, respectively; CK, no addition of dissolved organic matter. Module1-Module5 (M1-M5) represents the five modules with the highest number of nodes in each network. The five modules are displayed in different colors, and other modules are displayed in grey. The percentage represents the number of nodes in each module as a proportion of all nodes.

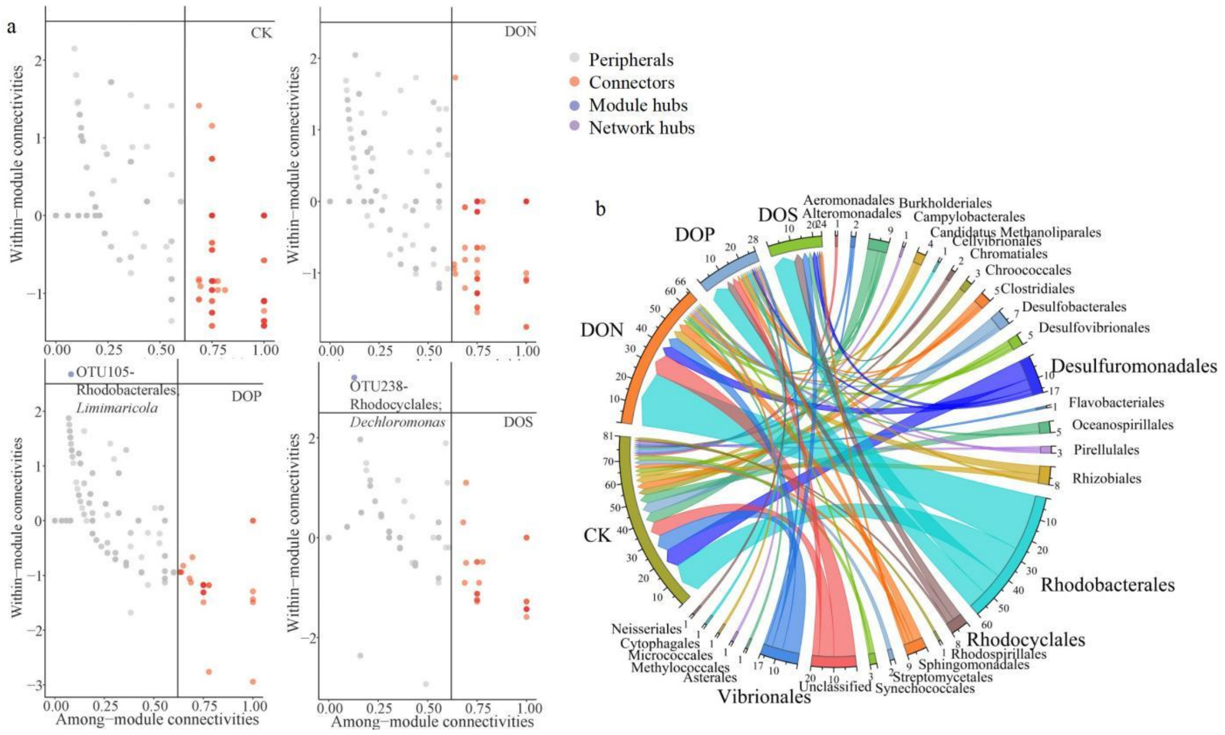

Fig. S5 Zi-Pi plot showing the distribution of all operational taxonomic units (OTUs) based on their topological properties. (a) Each dot symbol represents an OTU. All dots were distributed among the following four subcategories: peripherals (grey), connectors (red), module hubs (light blue), and network hubs (light purple). (b) Species composition of the connectors at the order level. CK, no DOM addition; DON, dissolved organic nitrogen; DOP, dissolved organic phosphorus; DOS, dissolved organic sulfur; OTU, operational taxonomic unit.

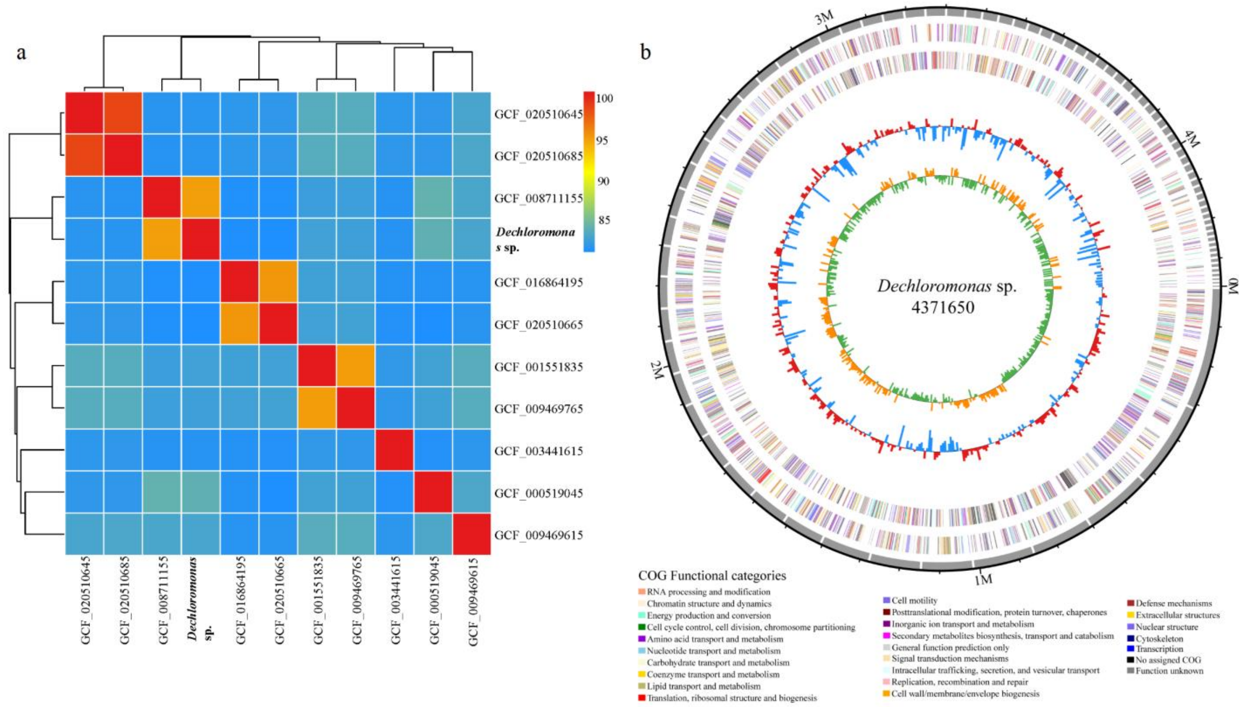

Fig. S6 Genomic analysis of *Dechloromonas* sp. (a) Average nucleotide identity heatmap between *Dechloromonas* sp. and other strains of the same genus. (b) Genetic map of the genomes of *Dechloromonas* sp. The rings 1-5 (from outside to inside) represent genome size, coding sequences (CDS) of the positive strand and negative strands (functional classification of COG with different colors), GC content, and GC skew.

Table S1. The excitation and emission wavelengths of four fluorescent components in chromophoric dissolved organic matter (CDOM) in the water column above the Haima cold seep.

Components	Exmax (nm)	Emmax(nm)	Description and source	Reference
C1	280	300-350	Biological production；Freshly production；Protein-like, Amino acid-like	(4)
C2	280	375	Mixture of polycyclic aromatic hydrocarbons (PAH) and aromatic amino acids	(5)
C3	220	340-360	Tryptophan-like	(6)
C4	230	430-475	Humic-like	

Table S2. Topological properties of diazotroph community co-occurrence networks based on the *nifH* gene.

Group	Nodes	Total edges	Positive edges	Negative edges	Network density	Average Path length	Clustering Coefficient
CK	296	1771	1647 (93.00%)	124 (7%)	0.041	4.528	0.75
DON	230	999	874 (87.49%)	125 (12.51%)	0.038	3.937	0.677
DOP	347	4019	3986 (99.18%)	33 (0.82%)	0.067	4.117	0.818
DOS	125	603	543 (90.05%)	60 (9.95%)	0.078	4.212	0.649

Table S3. Distributions of key functional genes (nitrogen and sulfur metabolism) in the metagenomes.

**Step**	**Substep**	**KO**	**Gene**	**R12-U**	**R345-U**	**R12-B**	**R345-B**
Dissimilatory nitrate reduction	Nitrate => Nitrite	K00370	narG | nitrate reductase alpha subunit	65.78	76.46	85.30	88.97
		K00371	narH | nitrate reductase beta subunit	52.44	49.22	63.01	61.40
		K00374	narI | nitrate reductase gamma subunit	25.77	24.23	32.31	37.54
		K02567	napA | periplasmic nitrate reductase NapA	4.29	11.71	36.48	24.88
		K02568	napB | cytochrome c-type protein NapB	4.03	12.58	25.27	16.94
	Nitrite => Ammonia	K00362	nirB | nitrite reductase (NADH) large subunit	87.92	101.75	99.42	149.81
		K00363	nirD | nitrite reductase (NADH) small subunit	58.85	70.53	62.48	94.98
		K03385	nrfA | nitrite reductase (cytochrome c-552)	0.13	0.18	1.68	1.48
		K15876	nrfH | cytochrome c nitrite reductase small subunit	0.13	0.27	0.34	0.65
Assimilatory nitrate reduction	Nitrate => Nitrite	K00367	narB | ferredoxin-nitrate reductase	1.57	0.91	4.09	8.36
		K00372	nasA | assimilatory nitrate reductase catalytic subunit	61.82	70.51	71.08	104.41
	Nitrite => Ammonia	K00366	nirA | ferredoxin-nitrite reductase	1.88	1.73	3.52	4.92
Denitrification	Nitrite => Nitric oxide	K00368	nirK | nitrite reductase (NO-forming)	52.93	47.76	52.54	31.72
		K15864	nirS | nitrite reductase (NO-forming) / hydroxylamine reductase	3.51	6.78	26.16	19.62
	Nitric oxide =>	K04561	norB | nitric oxide reductase subunit B	8.14	14.20	42.60	34.21

	Nitrous oxide						
		K02305	norC | nitric oxide reductase subunit C	6.29	5.28	26.85	17.20
	Nitrous oxide => Nitrogen	K00376	nosZ | nitrous-oxide reductase	14.79	11.64	36.41	30.57
Nitrogen fixation	Nitrogen => Ammonina	K02588	nifH | nitrogenase iron protein NifH	0.15	3.07	8.09	6.55
		K02586	nifD | nitrogenase molybdenum-iron protein alpha chain	0.30	4.66	11.89	8.56
		K02591	nifK | nitrogenase molybdenum-iron protein beta chain	0.00	2.09	10.12	7.83
Nitrification	Ammonia => Hydroxylamine	K10944	pmoA-amoA | methane/ammonia monooxygenase subunit A	56.13	42.33	37.83	19.14
		K10945	pmoB-amoB | methane/ammonia monooxygenase subunit B	63.81	47.69	40.83	23.92
		K10946	pmoC-amoC | methane/ammonia monooxygenase subunit C	72.65	50.84	69.66	32.78
	Hydroxylamine => Ammonia	K05601	hcp | hydroxylamine reductase	0.00	0.00	3.16	1.49
		K15864	nirS | nitrite reductase (NO-forming) / hydroxylamine reductase	3.51	6.78	26.16	19.62
	Nitrite => Nitrate	K00370	narG | nitrate reductase alpha subunit	65.78	76.46	85.30	88.97
		K00371	narH | nitrate reductase beta subunit	52.44	49.22	63.01	61.40
Nitrogen assimilation	Ammonia =>	K01915	glnA, GLUL | glutamine synthetase	1412.45	1223.59	1101.02	797.91

	Organic-N						
		K00265	gltB | glutamate synthase (NADPH/NADH) large chain	55.99	89.83	76.91	128.72
		K00266	gltD | glutamate synthase (NADPH/NADH) small chain	132.32	140.07	158.70	200.20
Nitrogen mineralization	Organ-N => ammonia	K15371	GDH2 | glutamate dehydrogenase	231.91	266.19	193.21	342.25
		K00261	GLUD1_2, gdhA | glutamate dehydrogenase (NAD(P)+)	8.87	8.15	35.73	26.21
		K00262	gdhA | glutamate dehydrogenase (NADP+)	71.18	76.47	122.56	123.57
		K01455	E3.5.1.49 | formamidase	38.72	54.89	57.65	51.03
		K01501	E3.5.5.1 | nitrilase	6.92	11.90	6.40	10.25
		K01725	cynS | cyanate lyase	52.20	66.70	46.49	68.28
		K00926	arcC | carbamate kinase	96.33	72.21	121.59	53.54
		K00549	metE | 5-methyltetrahydropteroyltriglutamate--homocysteine methyltransferase	406.34	393.81	531.14	342.33
Assimilatory sulfate reduction	Sulfate=> APS	K00956	cysN | sulfate adenylyltransferase subunit 1	33.25	37.97	49.57	72.94
		K00957	cysD | sulfate adenylyltransferase subunit 2	159.58	174.93	158.90	198.42
		K00955	cysNC | bifunctional enzyme CysN/CysC	73.50	95.90	50.42	92.72
	APS=> PAPS	K00955	cysNC | bifunctional enzyme CysN/CysC	73.50	95.90	50.42	92.72
		K00860	cysC | adenylylsulfate kinase	39.97	41.04	41.16	69.43
	PAPS=> Sulfite	K00390	cysH | phosphoadenosine phosphosulfate reductase	120.35	137.13	143.89	177.23
	Sulfite=> Sulfide	K00380	cysJ | sulfite reductase (NADPH) flavoprotein alpha-component	17.56	19.80	25.66	46.22

		K00381	cysI | sulfite reductase (NADPH) hemoprotein beta-component	92.86	91.61	95.00	150.01
		K00392	sir | sulfite reductase (ferredoxin)	1.85	1.04	2.67	11.58
Dissimilatory sulfate reduction and oxidation	Sulfate<=> APS	K00958	sat, met3 | sulfate adenylyltransferase	113.71	77.08	114.45	63.87
		K00988	APA1_2 | ATP adenylyltransferase	1.36	0.54	1.41	7.48
	APS<=> Sulfite	K00394	aprA | adenylylsulfate reductase, subunit A	25.55	18.91	40.93	14.46
		K00395	aprB | adenylylsulfate reductase, subunit B	79.12	66.91	78.36	36.50
	Sulfite<=> Sulfide	K11180	dsrA | sulfite reductase alpha subunit	10.25	7.97	6.62	1.94
		K11181	dsrB | sulfite reductase beta subunit	9.26	7.39	6.13	1.60
SOX system	thisulfate => sulfate	K17222	soxA | sulfur-oxidizing protein SoxA	14.69	31.60	25.13	27.93
		K17223	soxX | sulfur-oxidizing protein SoxX	22.10	36.84	23.66	31.81
		K17224	soxB | sulfur-oxidizing protein SoxB	19.39	25.41	24.06	21.34
		K17225	soxC | sulfane dehydrogenase subunit SoxC	13.01	22.52	30.47	24.13
		K17226	soxY | sulfur-oxidizing protein SoxY	47.63	73.62	86.87	68.75
		K17227	soxZ | sulfur-oxidizing protein SoxZ	22.86	41.11	39.90	36.14
Sulfide cycling	Sulfide=> Sulfur	K17229	fccB | sulfide dehydrogenase [flavocytochrome c] flavoprotein chain	5.39	17.36	25.06	18.01
		K17230	fccA | cytochrome subunit of sulfide dehydrogenase	23.25	51.24	43.06	52.89
	Sulfide=> (Sulfide)n	K17218	sqr | sulfide:quinone oxidoreductase	39.77	40.06	51.98	90.39
Sulfur mineralization	Organ-S => Sulfite	K11180	dsrA | sulfite reductase alpha subunit	10.25	7.97	6.62	1.94
		K11181	dsrB | sulfite reductase beta subunit	9.26	7.39	6.13	1.60
		K04091	ssuD | alkanesulfonate monooxygenase	19.73	51.75	27.39	32.46

		K00299	ssuE | FMN reductase	7.99	20.64	9.15	16.61
		K17228	sfnG | FMNH2-dependent dimethyl sulfone monooxygenase	2.61	10.01	2.20	4.69
		K16968	msmA | methanesulfonate monooxygenase large subunit	0.10	0.06	0.35	0.53
		K16969	msmB | methanesulfonate monooxygenase small subunit	0.21	0.07	1.24	0.93
		K15762	tmoC, tbuB, touC | toluene monooxygenase system ferredoxin subunit	2.23	2.03	1.59	1.49
		K15765	tmoF, tbuC, touF | toluene monooxygenase electron transfer component	0.38	1.23	0.72	0.60
		K03119	tauD | taurine dioxygenase	42.09	83.44	44.11	37.21
		K00456	CDO1 | cysteine dioxygenase	34.67	26.76	35.47	12.23
Sulfur assimilation	Sulfide=> Organ-S	K01739	metB | cystathionine gamma-synthase	0.57	0.32	0.86	1.02
		K10764	metZ | O-succinylhomoserine sulfhydrylase	71.91	91.59	75.29	110.23
		K01738	cysK | cysteine synthase A	138.43	168.09	155.50	241.40

Table S4. tRNAs identified in the genome of *Dechloromonas* sp..

Sequence Name	tRNA #	tRNA Bounds Begin	tRNA Bounds End	tRNA Type	Anti Codon	Intron Bounds Begin	Intron Bounds End	Inf Score
Dechloromonas sp.|RoV__7848	1	308539	308615	Ile2	CAT	0	0	84.3
Dechloromonas sp.|RoV__7848	2	190789	190713	Met	CAT	0	0	76.2
Dechloromonas sp.|RoV__11635	1	8124	8199	Ala	GGC	0	0	74.7
Dechloromonas sp.|RoV__11635	2	8223	8298	Glu	TTC	0	0	63.3
Dechloromonas sp.|RoV__11635	3	6488	6414	Val	CAC	0	0	78.7
Dechloromonas sp.|RoV__34559	1	3126	3051	Lys	TTT	0	0	95.1
Dechloromonas sp.|RoV__34559	2	3031	2956	Lys	CTT	0	0	88.5
Dechloromonas sp.|RoV__34559	3	2871	2796	fMet	CAT	0	0	84.9
Dechloromonas sp.|RoV__42763	1	59768	59852	Leu	CAA	0	0	76.7
Dechloromonas sp.|RoV__42763	2	37580	37496	Leu	GAG	0	0	62
Dechloromonas sp.|RoV__45419	1	2439	2523	Leu	CAG	0	0	69.2
Dechloromonas sp.|RoV__56667	1	7536	7611	His	GTG	0	0	73.2
Dechloromonas sp.|RoV__56667	2	1106	1031	Val	TAC	0	0	83

Dechloromonas sp.|RoV__103334	1	14603 3	146128	SeC	TCA	0	0	116.4
Dechloromonas sp.|RoV__125393	1	51649	51733	Leu	TAG	0	0	73.4
Dechloromonas sp.|RoV__126297	1	33701	33788	Ser	CGA	0	0	85.9
Dechloromonas sp.|RoV__134821	1	2856	2783	Gly	CCC	0	0	75.5
Dechloromonas sp.|RoV__142884	1	63998	63924	Thr	GGT	0	0	82.3
Dechloromonas sp.|RoV__174706	1	55004	54929	Phe	GAA	0	0	83.7
Dechloromonas sp.|RoV__249609	1	2099	2024	Gly	GCC	0	0	83.4
Dechloromonas sp.|RoV__249609	2	1959	1886	Cys	GCA	0	0	66.9
Dechloromonas sp.|RoV__249609	3	1877	1802	Gly	GCC	0	0	83.4
Dechloromonas sp.|RoV__249609	4	1731	1646	Leu	TAA	0	0	75
Dechloromonas sp.|RoV__255023	1	6044	6120	Pro	CGG	0	0	68.3
Dechloromonas sp.|RoV__272918	1	7161	7077	Leu	CAG	0	0	69.2
Dechloromonas sp.|RoV__282374	1	11154	11060	Leu	TAG	0	0	22
Dechloromonas sp.|RoV__282374	2	7880	7804	Val	GAC	0	0	82.1
Dechloromonas sp.|RoV__282374	3	273	197	Pro	GGG	0	0	72.9

Dechloromonas sp.|RoV__310962	1	1950	1877	Thr	TGT	0	0	58.2
Dechloromonas sp.|RoV__310962	2	277	202	fMet	CAT	0	0	84.9
Dechloromonas sp.|RoV__311770	1	18171	18096	Asn	GTT	0	0	75.5
Dechloromonas sp.|RoV__319519	1	40930	40838	Ser	GCT	0	0	91.1
Dechloromonas sp.|RoV__319519	2	13791	13715	Pro	TGG	0	0	79.4
Dechloromonas sp.|RoV__319519	3	13692	13616	Arg	TCT	0	0	84.5
Dechloromonas sp.|RoV__354008	1	133270	133344	Arg	CCT	0	0	57.8
Dechloromonas sp.|RoV__354008	2	117745	117658	Ser	TGA	0	0	83.6
Dechloromonas sp.|RoV__354008	3	33566	33482	Leu	GAG	0	0	65.1
Dechloromonas sp.|RoV__366872	1	62232	62308	Arg	CCG	0	0	78.8
Dechloromonas sp.|RoV__366872	2	73766	73690	Gln	TTG	0	0	76.5
Dechloromonas sp.|RoV__383859	1	127597	127687	Ser	GGA	0	0	85.5
Dechloromonas sp.|RoV__410737	1	28448	28373	Asn	GTT	0	0	75.5
Dechloromonas sp.|RoV__410737	2	3669	3593	Arg	ACG	0	0	81.1
Dechloromonas sp.|RoV__410737	3	3547	3471	Arg	ACG	0	0	81.1

Dechloromonas sp.|RoV__417217	1	65526	65601	Thr	CGT	0	0	85.3
Dechloromonas sp.|RoV__417827	1	3682	3607	Ala	CGC	0	0	77.4
Dechloromonas sp.|RoV__431861	1	16140	16216	Asp	GTC	0	0	89.1

Table S5. Kyoto Encyclopedia of Genes and Genome annotation of a metagenome-assembled genomes belonging to *Dechloromonas* sp..

Entry	Description
K01682	acnB; aconitate hydratase 2 / 2-methylisocitrate dehydratase [EC:4.2.1.3 4.2.1.99]
K14260	alaA; alanine-synthesizing transaminase [EC:2.6.1.66 2.6.1.2]
K14682	argAB; amino-acid N-acetyltransferase [EC:2.3.1.1]
K00930	argB; acetylglutamate kinase [EC:2.7.2.8]
K00145	argC; N-acetyl-gamma-glutamyl-phosphate reductase [EC:1.2.1.38]
K00821	argD; acetylornithine/N-succinyldiaminopimelate aminotransferase [EC:2.6.1.11 2.6.1.17]
K00620	argJ; glutamate N-acetyltransferase / amino-acid N-acetyltransferase [EC:2.3.1.35 2.3.1.1]
K00133	asd; aspartate-semialdehyde dehydrogenase [EC:1.2.1.11]
K01647	CS, gltA; citrate synthase [EC:2.3.3.1]
K01652	E2.2.1.6L, ilvB, ilvG, ilvI; acetolactate synthase I/II/III large subunit [EC:2.2.1.6]
K01653	E2.2.1.6S, ilvH, ilvN; acetolactate synthase I/III small subunit [EC:2.2.1.6]
K00826	E2.6.1.42, ilvE; branched-chain amino acid aminotransferase [EC:2.6.1.42]
K00031	IDH1, IDH2, icd; isocitrate dehydrogenase [EC:1.1.1.42]
K00053	ilvC; ketol-acid reductoisomerase [EC:1.1.1.86]
K01687	ilvD; dihydroxy-acid dehydratase [EC:4.2.1.9]
K01649	leuA, IMS; 2-isopropylmalate synthase [EC:2.3.3.13]
K00052	leuB, IMDH; 3-isopropylmalate dehydrogenase [EC:1.1.1.85]
K01703	leuC, IPMI-L; 3-isopropylmalate/(R)-2-methylmalate dehydratase large subunit [EC:4.2.1.33 4.2.1.35]
K01704	leuD, IPMI-S; 3-isopropylmalate/(R)-2-methylmalate dehydratase small subunit [EC:4.2.1.33 4.2.1.35]
K00928	lysC; aspartate kinase [EC:2.7.2.4]
K09691	ABC-2.LPSE.A; lipopolysaccharide transport system ATP-binding protein
K09690	ABC-2.LPSE.P; lipopolysaccharide transport system permease protein
K02012	afuA, fbpA; iron(III) transport system substrate-binding protein
K02011	afuB, fbpB; iron(III) transport system permease protein
K02010	afuC, fbpC; iron(III) transport system ATP-binding protein [EC:7.2.2.7]
K06858	btuF; vitamin B12 transport system substrate-binding protein

K02007	cbiM; cobalt/nickel transport system permease protein
K02009	cbiN; cobalt/nickel transport protein
K02006	cbiO; cobalt/nickel transport system ATP-binding protein
K02193	ccmA; heme exporter protein A [EC:7.6.2.5]
K02194	ccmB; heme exporter protein B
K02195	ccmC; heme exporter protein C
K02196	ccmD; heme exporter protein D
K02045	cysA; sulfate/thiosulfate transport system ATP-binding protein [EC:7.3.2.3]
K02046	cysU; sulfate/thiosulfate transport system permease protein
K02047	cysW; sulfate/thiosulfate transport system permease protein
K09812	ftsE; cell division transport system ATP-binding protein
K09811	ftsX; cell division transport system permease protein
K10001	gltI, aatJ; glutamate/aspartate transport system substrate-binding protein
K10003	gltJ, aatQ; glutamate/aspartate transport system permease protein
K10002	gltK, aatM; glutamate/aspartate transport system permease protein
K10004	gltL, aatP; glutamate/aspartate transport system ATP-binding protein [EC:7.4.2.1]
K12541	lapB; ATP-binding cassette, subfamily C, bacterial LapB
K01996	livF; branched-chain amino acid transport system ATP-binding protein
K01995	livG; branched-chain amino acid transport system ATP-binding protein
K01997	livH; branched-chain amino acid transport system permease protein
K01999	livK; branched-chain amino acid transport system substrate-binding protein
K01998	livM; branched-chain amino acid transport system permease protein
K09808	lolC_E; lipoprotein-releasing system permease protein
K09810	lolD; lipoprotein-releasing system ATP-binding protein [EC:3.6.3.-]
K06861	lptB; lipopolysaccharide export system ATP-binding protein [EC:3.6.3.-]
K07091	lptF; lipopolysaccharide export system permease protein
K11720	lptG; lipopolysaccharide export system permease protein
K07122	mlaB; phospholipid transport system transporter-binding protein
K07323	mlaC; phospholipid transport system substrate-binding protein
K02067	mlaD, linM; phospholipid/cholesterol/gamma-HCH transport system substrate-binding protein
K02066	mlaE, linK; phospholipid/cholesterol/gamma-HCH transport system permease protein

K02065	mlaF, linL, mkl; phospholipid/cholesterol/gamma-HCH transport system ATP-binding protein
K02020	modA; molybdate transport system substrate-binding protein
K02018	modB; molybdate transport system permease protein
K02017	modC; molybdate transport system ATP-binding protein [EC:7.3.2.5]
K11085	msbA; ATP-binding cassette, subfamily B, bacterial MsbA [EC:3.6.3.-]
K09695	nodI; lipooligosaccharide transport system ATP-binding protein
K09694	nodJ; lipooligosaccharide transport system permease protein
K15576	nrtA, nasF, cynA; nitrate/nitrite transport system substrate-binding protein
K15577	nrtB, nasE, cynB; nitrate/nitrite transport system permease protein
K15578	nrtC, nasD; nitrate/nitrite transport system ATP-binding protein [EC:3.6.3.-]
K02041	phnC; phosphonate transport system ATP-binding protein [EC:7.3.2.2]
K02044	phnD; phosphonate transport system substrate-binding protein
K02042	phnE; phosphonate transport system permease protein
K02002	proX; glycine betaine/proline transport system substrate-binding protein
K02038	pstA; phosphate transport system permease protein
K02036	pstB; phosphate transport system ATP-binding protein [EC:7.3.2.1]
K02037	pstC; phosphate transport system permease protein
K02040	pstS; phosphate transport system substrate-binding protein
K23163	sbp; sulfate/thiosulfate transport system substrate-binding protein
K15553	ssuA; sulfonate transport system substrate-binding protein
K15555	ssuB; sulfonate transport system ATP-binding protein [EC:3.6.3.-]
K15554	ssuC; sulfonate transport system permease protein
K11959	urtA; urea transport system substrate-binding protein
K11960	urtB; urea transport system permease protein
K11961	urtC; urea transport system permease protein
K11962	urtD; urea transport system ATP-binding protein
K11963	urtE; urea transport system ATP-binding protein
K00973	rfbA, rmlA, rffH; glucose-1-phosphate thymidylyltransferase [EC:2.7.7.24]
K01710	rfbB, rmlB, rffG; dTDP-glucose 4,6-dehydratase [EC:4.2.1.46]
K01897	ACSL, fadD; long-chain acyl-CoA synthetase [EC:6.2.1.3]
K01596	E4.1.1.32, pckA, PCK; phosphoenolpyruvate carboxykinase (GTP) [EC:4.1.1.32]

K01940	argG, ASS1; argininosuccinate synthase [EC:6.3.4.5]
K01755	argH, ASL; argininosuccinate lyase [EC:4.3.2.1]
K01953	asnB, ASNS; asparagine synthase (glutamine-hydrolysing) [EC:6.3.5.4]
K01744	aspA; aspartate ammonia-lyase [EC:4.3.1.1]
K05597	aspQ, ansB, ansA; glutamin-(asparagin-)ase [EC:3.5.1.38]
K01956	carA, CPA1; carbamoyl-phosphate synthase small subunit [EC:6.3.5.5]
K01955	carB, CPA2; carbamoyl-phosphate synthase large subunit [EC:6.3.5.5]
K00262	E1.4.1.4, gdhA; glutamate dehydrogenase (NADP+) [EC:1.4.1.4]
K00135	gabD; succinate-semialdehyde dehydrogenase / glutarate-semialdehyde dehydrogenase [EC:1.2.1.16 1.2.1.79 1.2.1.20]
K00820	glmS, GFPT; glutamine---fructose-6-phosphate transaminase (isomerizing) [EC:2.6.1.16]
K01915	glnA, GLUL; glutamine synthetase [EC:6.3.1.2]
K00265	gltB; glutamate synthase (NADPH) large chain [EC:1.4.1.13]
K00266	gltD; glutamate synthase (NADPH) small chain [EC:1.4.1.13]
K00261	GLUD1_2, gdhA; glutamate dehydrogenase (NAD(P)+) [EC:1.4.1.3]
K00278	nadB; L-aspartate oxidase [EC:1.4.3.16]
K01939	purA, ADSS; adenylosuccinate synthase [EC:6.3.4.4]
K01756	purB, ADSL; adenylosuccinate lyase [EC:4.3.2.2]
K00764	purF, PPAT; amidophosphoribosyltransferase [EC:2.4.2.14]
K13821	putA; RHH-type transcriptional regulator, proline utilization regulon repressor / proline dehydrogenase / delta 1-pyrroline-5-carboxylate dehydrogenase [EC:1.5.5.2 1.2.1.88]
K00609	pyrB, PYR2; aspartate carbamoyltransferase catalytic subunit [EC:2.1.3.2]
K00632	fadA, fadI; acetyl-CoA acyltransferase [EC:2.3.1.16]
K16011	algA, xanB, rfbA, wbpW, pslB; mannose-1-phosphate guanylyltransferase / mannose-6-phosphate isomerase [EC:2.7.7.13 5.3.1.8]
K07102	amgK; N-acetylmuramate 1-kinase [EC:2.7.1.221]
K12452	ascC, ddhC, rfbH; CDP-4-dehydro-6-deoxyglucose reductase, E1 [EC:1.17.1.1]
K00523	ascD, ddhD, rfbI; CDP-4-dehydro-6-deoxyglucose reductase, E3 [EC:1.17.1.1]
K08679	E5.1.3.6; UDP-glucuronate 4-epimerase [EC:5.1.3.6]
K01784	galE, GALE; UDP-glucose 4-epimerase [EC:5.1.3.2]
K03431	glmM; phosphoglucosamine mutase [EC:5.4.2.10]
K04042	glmU; bifunctional UDP-N-acetylglucosamine pyrophosphorylase / Glucosamine-1-phosphate N-acetyltransferase [EC:2.7.7.23 2.3.1.157]

K01810	GPI, pgi; glucose-6-phosphate isomerase [EC:5.3.1.9]
K22292	mupP; N-acetyl-D-muramate 6-phosphate phosphatase [EC:3.1.3.105]
K00790	murA; UDP-N-acetylglucosamine 1-carboxyvinyltransferase [EC:2.5.1.7]
K00075	murB; UDP-N-acetylmuramate dehydrogenase [EC:1.3.1.98]
K00992	murU; N-acetyl-alpha-D-muramate 1-phosphate uridylyltransferase [EC:2.7.7.99]
K01207	nagZ; beta-N-acetylhexosaminidase [EC:3.2.1.52]
K15778	pmm-pgm; phosphomannomutase / phosphoglucomutase [EC:5.4.2.8 5.4.2.2]
K00978	rfbF; glucose-1-phosphate cytidylyltransferase [EC:2.7.7.33]
K01709	rfbG; CDP-glucose 4,6-dehydratase [EC:4.2.1.45]
K00012	UGDH, ugd; UDPglucose 6-dehydrogenase [EC:1.1.1.22]
K00963	UGP2, galU, galF; UTP--glucose-1-phosphate uridylyltransferase [EC:2.7.7.9]
K02474	wbpO; UDP-N-acetyl-D-galactosamine dehydrogenase [EC:1.1.1.-]
K01791	wecB; UDP-N-acetylglucosamine 2-epimerase (non-hydrolysing) [EC:5.1.3.14]
K01872	AARS, alaS; alanyl-tRNA synthetase [EC:6.1.1.7]
K01876	aspS; aspartyl-tRNA synthetase [EC:6.1.1.12]
K01883	CARS, cysS; cysteinyl-tRNA synthetase [EC:6.1.1.16]
K01885	EARS, gltX; glutamyl-tRNA synthetase [EC:6.1.1.17]
K01889	FARSA, pheS; phenylalanyl-tRNA synthetase alpha chain [EC:6.1.1.20]
K01890	FARSB, pheT; phenylalanyl-tRNA synthetase beta chain [EC:6.1.1.20]
K02433	gatA, QRSL1; aspartyl-tRNA(Asn)/glutamyl-tRNA(Gln) amidotransferase subunit A [EC:6.3.5.6 6.3.5.7]
K02434	gatB, PET112; aspartyl-tRNA(Asn)/glutamyl-tRNA(Gln) amidotransferase subunit B [EC:6.3.5.6 6.3.5.7]
K02435	gatC, GATC; aspartyl-tRNA(Asn)/glutamyl-tRNA(Gln) amidotransferase subunit C [EC:6.3.5.6 6.3.5.7]
K01878	glyQ; glycyl-tRNA synthetase alpha chain [EC:6.1.1.14]
K01879	glyS; glycyl-tRNA synthetase beta chain [EC:6.1.1.14]
K01892	HARS, hisS; histidyl-tRNA synthetase [EC:6.1.1.21]
K01870	IARS, ileS; isoleucyl-tRNA synthetase [EC:6.1.1.5]
K04567	KARS, lysS; lysyl-tRNA synthetase, class II [EC:6.1.1.6]
K01869	LARS, leuS; leucyl-tRNA synthetase [EC:6.1.1.4]
K01874	MARS, metG; methionyl-tRNA synthetase [EC:6.1.1.10]
K00604	MTFMT, fmt; methionyl-tRNA formyltransferase [EC:2.1.2.9]
K01881	PARS, proS; prolyl-tRNA synthetase [EC:6.1.1.15]

K01886	QARS, glnS; glutaminyl-tRNA synthetase [EC:6.1.1.18]
K01887	RARS, argS; arginyl-tRNA synthetase [EC:6.1.1.19]
K01875	SARS, serS; seryl-tRNA synthetase [EC:6.1.1.11]
K01042	selA; L-seryl-tRNA(Ser) seleniumtransferase [EC:2.9.1.1]
K01868	TARS, thrS; threonyl-tRNA synthetase [EC:6.1.1.3]
K01873	VARS, valS; valyl-tRNA synthetase [EC:6.1.1.9]
K01867	WARS, trpS; tryptophanyl-tRNA synthetase [EC:6.1.1.2]
K01866	YARS, tyrS; tyrosyl-tRNA synthetase [EC:6.1.1.1]
K01512	acyP; acylphosphatase [EC:3.6.1.7]
K01692	paaF, echA; enoyl-CoA hydratase [EC:4.2.1.17]
K03186	ubiX, bsdB, PAD1; flavin prenyltransferase [EC:2.5.1.129]
K03841	FBP, fbp; fructose-1,6-bisphosphatase I [EC:3.1.3.11]
K01578	MLYCD; malonyl-CoA decarboxylase [EC:4.1.1.9]
K04079	HSP90A, htpG; molecular chaperone HtpG
K08738	CYC; cytochrome c
K03386	PRDX2_4, ahpC; peroxiredoxin (alkyl hydroperoxide reductase subunit C) [EC:1.11.1.15]
K00432	gpx, btuE, bsaA; glutathione peroxidase [EC:1.11.1.9]
K01584	adiA; arginine decarboxylase [EC:4.1.1.19]
K00824	dat; D-alanine transaminase [EC:2.6.1.21]
K01470	E3.5.2.10; creatinine amidohydrolase [EC:3.5.2.10]
K01476	E3.5.3.1, rocF, arg; arginase [EC:3.5.3.1]
K01750	E4.3.1.12, ocd; ornithine cyclodeaminase [EC:4.3.1.12]
K03365	FCY1; cytosine/creatinine deaminase [EC:3.5.4.1 3.5.4.21]
K01259	pip; proline iminopeptidase [EC:3.4.11.5]
K00147	proA; glutamate-5-semialdehyde dehydrogenase [EC:1.2.1.41]
K00931	proB; glutamate 5-kinase [EC:2.7.2.11]
K00286	proC; pyrroline-5-carboxylate reductase [EC:1.5.1.2]
K09471	puuB, ordL; gamma-glutamylputrescine oxidase [EC:1.4.3.-]
K01611	speD, AMD1; S-adenosylmethionine decarboxylase [EC:4.1.1.50]
K00797	speE, SRM, SPE3; spermidine synthase [EC:2.5.1.16]
K00611	OTC, argF, argI; ornithine carbamoyltransferase [EC:2.1.3.3]

K01430	ureA; urease subunit gamma [EC:3.5.1.5]
K01429	ureB; urease subunit beta [EC:3.5.1.5]
K01428	ureC; urease subunit alpha [EC:3.5.1.5]
K03776	aer; aerotaxis receptor
K03407	cheA; two-component system, chemotaxis family, sensor kinase CheA [EC:2.7.13.3]
K03412	cheB; two-component system, chemotaxis family, protein-glutamate methylesterase/glutaminase [EC:3.1.1.61 3.5.1.44]
K03411	cheD; chemotaxis protein CheD [EC:3.5.1.44]
K00575	cheR; chemotaxis protein methyltransferase CheR [EC:2.1.1.80]
K03415	cheV; two-component system, chemotaxis family, chemotaxis protein CheV
K03408	cheW; purine-binding chemotaxis protein CheW
K03413	cheY; two-component system, chemotaxis family, chemotaxis protein CheY
K03414	cheZ; chemotaxis protein CheZ
K02410	fliG; flagellar motor switch protein FliG
K02416	fliM; flagellar motor switch protein FliM
K02417	fliNY, fliN; flagellar motor switch protein FliN/FliY
K03406	mcp; methyl-accepting chemotaxis protein
K02556	motA; chemotaxis protein MotA
K02557	motB; chemotaxis protein MotB
K03110	ftsY; fused signal recognition particle receptor
K02453	gspD; general secretion pathway protein D
K02454	gspE; general secretion pathway protein E [EC:7.4.2.8]
K02456	gspG; general secretion pathway protein G
K11891	impL, vasK, icmF; type VI secretion system protein ImpL
K03070	secA; preprotein translocase subunit SecA [EC:7.4.2.8]
K03071	secB; preprotein translocase subunit SecB
K03072	secD; preprotein translocase subunit SecD
K03074	secF; preprotein translocase subunit SecF
K03075	secG; preprotein translocase subunit SecG
K03076	secY; preprotein translocase subunit SecY
K03106	SRP54, ffh; signal recognition particle subunit SRP54 [EC:3.6.5.4]
K03116	tatA; sec-independent protein translocase protein TatA

K03117	tatB; sec-independent protein translocase protein TatB
K03118	tatC; sec-independent protein translocase protein TatC
K12340	tolC; outer membrane protein
K11904	vgrG; type VI secretion system secreted protein VgrG
K03194	virB1; type IV secretion system protein VirB1
K03210	yajC; preprotein translocase subunit YajC
K03217	yidC, spoIIIJ, OXA1, ccfA; YidC/Oxa1 family membrane protein insertase
K01247	alkA; DNA-3-methyladenine glycosylase II [EC:3.2.2.21]
K01142	E3.1.11.2, xthA; exodeoxyribonuclease III [EC:3.1.11.2]
K01972	E6.5.1.2, ligA, ligB; DNA ligase (NAD+) [EC:6.5.1.2]
K10563	mutM, fpg; formamidopyrimidine-DNA glycosylase [EC:3.2.2.23 4.2.99.18]
K03575	mutY; A/G-specific adenine glycosylase [EC:3.2.2.31]
K10773	NTH; endonuclease III [EC:4.2.99.18]
K02335	polA; DNA polymerase I [EC:2.7.7.7]
K07462	recJ; single-stranded-DNA-specific exonuclease [EC:3.1.-.-]
K01246	tag; DNA-3-methyladenine glycosylase I [EC:3.2.2.20]
K21929	udg; uracil-DNA glycosylase [EC:3.2.2.27]
K05549	benA-xylX; benzoate/toluate 1,2-dioxygenase subunit alpha [EC:1.14.12.10 1.14.12.-]
K05550	benB-xylY; benzoate/toluate 1,2-dioxygenase subunit beta [EC:1.14.12.10 1.14.12.-]
K05784	benC-xylZ; benzoate/toluate 1,2-dioxygenase reductase component [EC:1.18.1.-]
K05783	benD-xylL; dihydroxycyclohexadiene carboxylate dehydrogenase [EC:1.3.1.25 1.3.1.-]
K18364	bphH, xylJ, tesE; 2-oxopent-4-enoate/cis-2-oxohex-4-enoate hydratase [EC:4.2.1.80 4.2.1.132]
K18365	bphI, xylK, nahM, tesG; 4-hydroxy-2-oxovalerate/4-hydroxy-2-oxohexanoate aldolase [EC:4.1.3.39 4.1.3.43]
K18366	bphJ, xylQ, nahO, tesF; acetaldehyde/propanal dehydrogenase [EC:1.2.1.10 1.2.1.87]
K00446	dmpB, xylE; catechol 2,3-dioxygenase [EC:1.13.11.2]
K10217	dmpC, xylG, praB; aminomuconate-semialdehyde/2-hydroxymuconate-6-semialdehyde dehydrogenase [EC:1.2.1.32 1.2.1.85]
K01617	dmpH, xylI, nahK; 2-oxo-3-hexenedioate decarboxylase [EC:4.1.1.77]
K16249	dmpK, poxA, tomA0; phenol/toluene 2-monooxygenase (NADH) P0/A0
K16243	dmpL, poxB, tomA1; phenol/toluene 2-monooxygenase (NADH) P1/A1 [EC:1.14.13.244 1.14.13.243]
K16244	dmpM, poxC, tomA2; phenol/toluene 2-monooxygenase (NADH) P2/A2 [EC:1.14.13.244 1.14.13.243]
K16242	dmpN, poxD, tomA3; phenol/toluene 2-monooxygenase (NADH) P3/A3 [EC:1.14.13.244 1.14.13.243]

K16245	dmpO, poxE, tomA4; phenol/toluene 2-monooxygenase (NADH) P4/A4 [EC:1.14.13.244 1.14.13.243]
K16246	dmpP, poxF, tomA5; phenol/toluene 2-monooxygenase (NADH) P5/A5 [EC:1.14.13.244 1.14.13.243]
K00626	E2.3.1.9, atoB; acetyl-CoA C-acetyltransferase [EC:2.3.1.9]
K01782	fadJ; 3-hydroxyacyl-CoA dehydrogenase / enoyl-CoA hydratase / 3-hydroxybutyryl-CoA epimerase [EC:1.1.1.35 4.2.1.17 5.1.2.3]
K00252	GCDH, gcdH; glutaryl-CoA dehydrogenase [EC:1.3.8.6]
K00074	paaH, hbd, fadB, mmgB; 3-hydroxybutyryl-CoA dehydrogenase [EC:1.1.1.157]
K01821	praC, xylH; 4-oxalocrotonate tautomerase [EC:5.3.2.6]
K01918	panC; pantoate--beta-alanine ligase [EC:6.3.2.1]
K01579	panD; aspartate 1-decarboxylase [EC:4.1.1.11]
K15777	DOPA; 4,5-DOPA dioxygenase extradiol [EC:1.13.11.-]
K10914	crp; CRP/FNR family transcriptional regulator, cyclic AMP receptor protein
K06204	dksA; DnaK suppressor protein
K07638	envZ; two-component system, OmpR family, osmolarity sensor histidine kinase EnvZ [EC:2.7.13.3]
K02398	flgM; negative regulator of flagellin synthesis FlgM
K02402	flhC; flagellar transcriptional activator FlhC
K02405	fliA; RNA polymerase sigma factor for flagellar operon FliA
K03566	gcvA; LysR family transcriptional regulator, glycine cleavage system transcriptional activator
K07659	ompR; two-component system, OmpR family, phosphate regulon response regulator OmpR
K04761	oxyR; LysR family transcriptional regulator, hydrogen peroxide-inducible genes activator
K07689	uvrY, gacA, varA; two-component system, NarL family, invasion response regulator UvrY
K01991	wza, gfcE; polysaccharide biosynthesis/export protein
K21087	ycgR; flagellar brake protein
K06596	chpA; chemosensory pili system protein ChpA (sensor histidine kinase/response regulator)
K23127	fimW; cyclic-di-GMP-binding protein
K20978	hsbA; HptB-dependent secretion and biofilm anti anti-sigma factor
K20977	hsbR; two-component system, HptB-dependent secretion and biofilm response regulator
K11902	impA; type VI secretion system protein ImpA
K11890	impM; type VI secretion system protein ImpM
K20974	K20974; two-component system, sensor histidine kinase [EC:2.7.13.3]
K21023	mucR; diguanylate cyclase [EC:2.7.7.65]
K02657	pilG; twitching motility two-component system response regulator PilG

K02658	pilH; twitching motility two-component system response regulator PilH
K02659	pilI; twitching motility protein PilI
K02660	pilJ; twitching motility protein PilJ
K13061	rhlI, phzI, solI, cepI, tofI; acyl homoserine lactone synthase [EC:2.3.1.184]
K01657	trpE; anthranilate synthase component I [EC:4.1.3.27]
K01658	trpG; anthranilate synthase component II [EC:4.1.3.27]
K00640	cysE; serine O-acetyltransferase [EC:2.3.1.30]
K03557	fis; Fis family transcriptional regulator, factor for inversion stimulation protein
K10942	flrB, fleS; two-component system, sensor histidine kinase FlrB [EC:2.7.13.3]
K10943	flrC, fleR; two-component system, response regulator FlrC
K03666	hfq; host factor-I protein
K10924	mshA; MSHA pilin protein MshA
K10926	mshC; MSHA pilin protein MshC
K10927	mshD; MSHA pilin protein MshD
K12276	mshE; MSHA biogenesis protein MshE
K01912	paaK; phenylacetate-CoA ligase [EC:6.2.1.30]
K03092	rpoN; RNA polymerase sigma-54 factor
K05946	tagA, tarA; N-acetylglucosaminyldiphosphoundecaprenol N-acetyl-beta-D-mannosaminyltransferase [EC:2.4.1.187]
K03606	wcaJ; putative colanic acid biosysnthesis UDP-glucose lipid carrier transferase
K00800	aroA; 3-phosphoshikimate 1-carboxyvinyltransferase [EC:2.5.1.19]
K01735	aroB; 3-dehydroquinate synthase [EC:4.2.3.4]
K01736	aroC; chorismate synthase [EC:4.2.3.5]
K00014	aroE; shikimate dehydrogenase [EC:1.1.1.25]
K00891	aroK, aroL; shikimate kinase [EC:2.7.1.71]
K03786	aroQ, qutE; 3-dehydroquinate dehydratase II [EC:4.2.1.10]
K01697	CBS; cystathionine beta-synthase [EC:4.2.1.22]
K01758	CTH; cystathionine gamma-lyase [EC:4.4.1.1]
K01738	cysK; cysteine synthase [EC:2.5.1.47]
K12339	cysM; S-sulfo-L-cysteine synthase (O-acetyl-L-serine-dependent) [EC:2.5.1.144]
K01714	dapA; 4-hydroxy-tetrahydrodipicolinate synthase [EC:4.3.3.7]
K00215	dapB; 4-hydroxy-tetrahydrodipicolinate reductase [EC:1.17.1.8]

K14267	dapC; N-succinyldiaminopimelate aminotransferase [EC:2.6.1.17]
K00674	dapD; 2,3,4,5-tetrahydropyridine-2,6-dicarboxylate N-succinyltransferase [EC:2.3.1.117]
K01439	dapE; succinyl-diaminopimelate desuccinylase [EC:3.5.1.18]
K01778	dapF; diaminopimelate epimerase [EC:5.1.1.7]
K00615	E2.2.1.1, tktA, tktB; transketolase [EC:2.2.1.1]
K01626	E2.5.1.54, aroF, aroG, aroH; 3-deoxy-7-phosphoheptulonate synthase [EC:2.5.1.54]
K01754	E4.3.1.19, ilvA, tdcB; threonine dehydratase [EC:4.3.1.19]
K01689	ENO, eno; enolase [EC:4.2.1.11]
K01624	FBA, fbaA; fructose-bisphosphate aldolase, class II [EC:4.1.2.13]
K00134	GAPDH, gapA; glyceraldehyde 3-phosphate dehydrogenase [EC:1.2.1.12]
K00600	glyA, SHMT; glycine hydroxymethyltransferase [EC:2.1.2.1]
K15634	gpmB; probable phosphoglycerate mutase [EC:5.4.2.12]
K15633	gpmI; 2,3-bisphosphoglycerate-independent phosphoglycerate mutase [EC:5.4.2.12]
K01814	hisA; phosphoribosylformimino-5-aminoimidazole carboxamide ribotide isomerase [EC:5.3.1.16]
K01693	hisB; imidazoleglycerol-phosphate dehydratase [EC:4.2.1.19]
K00817	hisC; histidinol-phosphate aminotransferase [EC:2.6.1.9]
K00013	hisD; histidinol dehydrogenase [EC:1.1.1.23]
K01523	hisE; phosphoribosyl-ATP pyrophosphohydrolase [EC:3.6.1.31]
K02500	hisF; imidazole glycerol-phosphate synthase subunit HisF [EC:4.3.2.10]
K00765	hisG; ATP phosphoribosyltransferase [EC:2.4.2.17]
K02501	hisH; imidazole glycerol-phosphate synthase subunit HisH [EC:4.3.2.10]
K01496	hisI; phosphoribosyl-AMP cyclohydrolase [EC:3.5.4.19]
K02502	hisZ; ATP phosphoribosyltransferase regulatory subunit
K00003	hom; homoserine dehydrogenase [EC:1.1.1.3]
K01586	lysA; diaminopimelate decarboxylase [EC:4.1.1.20]
K00548	metH, MTR; 5-methyltetrahydrofolate--homocysteine methyltransferase [EC:2.1.1.13]
K00789	metK; S-adenosylmethionine synthetase [EC:2.5.1.6]
K14155	patB, malY; cysteine-S-conjugate beta-lyase [EC:4.4.1.13]
K00927	PGK, pgk; phosphoglycerate kinase [EC:2.7.2.3]
K00873	PK, pyk; pyruvate kinase [EC:2.7.1.40]
K00948	PRPS, prsA; ribose-phosphate pyrophosphokinase [EC:2.7.6.1]

K01783	rpe, RPE; ribulose-phosphate 3-epimerase [EC:5.1.3.1]
K01807	rpiA; ribose 5-phosphate isomerase A [EC:5.3.1.6]
K01079	serB, PSPH; phosphoserine phosphatase [EC:3.1.3.3]
K02204	thrB2; homoserine kinase type II [EC:2.7.1.39]
K01733	thrC; threonine synthase [EC:4.2.3.1]
K02203	thrH; phosphoserine / homoserine phosphotransferase [EC:3.1.3.3 2.7.1.39]
K01803	TPI, tpiA; triosephosphate isomerase (TIM) [EC:5.3.1.1]
K01695	trpA; tryptophan synthase alpha chain [EC:4.2.1.20]
K01696	trpB; tryptophan synthase beta chain [EC:4.2.1.20]
K06001	trpB; tryptophan synthase beta chain [EC:4.2.1.20]
K01609	trpC; indole-3-glycerol phosphate synthase [EC:4.1.1.48]
K00766	trpD; anthranilate phosphoribosyltransferase [EC:2.4.2.18]
K01817	trpF; phosphoribosylanthranilate isomerase [EC:5.3.1.24]
K00832	tyrB; aromatic-amino-acid transaminase [EC:2.6.1.57]
K00249	ACADM, acd; acyl-CoA dehydrogenase [EC:1.3.8.7]
K01962	accA; acetyl-CoA carboxylase carboxyl transferase subunit alpha [EC:6.4.1.2 2.1.3.15]
K02160	accB, bccP; acetyl-CoA carboxylase biotin carboxyl carrier protein
K01961	accC; acetyl-CoA carboxylase, biotin carboxylase subunit [EC:6.4.1.2 6.3.4.14]
K01963	accD; acetyl-CoA carboxylase carboxyl transferase subunit beta [EC:6.4.1.2 2.1.3.15]
K00163	aceE; pyruvate dehydrogenase E1 component [EC:1.2.4.1]
K02078	acpP; acyl carrier protein
K01895	ACSS1_2, acs; acetyl-CoA synthetase [EC:6.2.1.1]
K13953	adhP; alcohol dehydrogenase, propanol-preferring [EC:1.1.1.1]
K00939	adk, AK; adenylate kinase [EC:2.7.4.3]
K00957	cysD; sulfate adenylyltransferase subunit 2 [EC:2.7.7.4]
K00956	cysN; sulfate adenylyltransferase subunit 1 [EC:2.7.7.4]
K00627	DLAT, aceF, pdhC; pyruvate dehydrogenase E2 component (dihydrolipoamide acetyltransferase) [EC:2.3.1.12]
K00382	DLD, lpd, pdhD; dihydrolipoamide dehydrogenase [EC:1.8.1.4]
K00658	DLST, sucB; 2-oxoglutarate dehydrogenase E2 component (dihydrolipoamide succinyltransferase) [EC:2.3.1.61]
K00099	dxr; 1-deoxy-D-xylulose-5-phosphate reductoisomerase [EC:1.1.1.267]
K01662	dxs; 1-deoxy-D-xylulose-5-phosphate synthase [EC:2.2.1.7]

K01092	E3.1.3.25, IMPA, suhB; myo-inositol-1(or 4)-monophosphatase [EC:3.1.3.25]
K01676	E4.2.1.2A, fumA, fumB; fumarate hydratase, class I [EC:4.2.1.2]
K01677	E4.2.1.2AA, fumA; fumarate hydratase subunit alpha [EC:4.2.1.2]
K01792	E5.1.3.15; glucose-6-phosphate 1-epimerase [EC:5.1.3.15]
K00114	exaA; alcohol dehydrogenase (cytochrome c) [EC:1.1.2.8]
K00645	fabD, MCAT, MCT1; [acyl-carrier-protein] S-malonyltransferase [EC:2.3.1.39]
K00059	fabG, OAR1; 3-oxoacyl-[acyl-carrier protein] reductase [EC:1.1.1.100]
K00208	fabI; enoyl-[acyl-carrier protein] reductase I [EC:1.3.1.9 1.3.1.10]
K11529	gck, gckA, GLYCTK; glycerate 2-kinase [EC:2.7.1.165]
K03526	gcpE, ispG; (E)-4-hydroxy-3-methylbut-2-enyl-diphosphate synthase [EC:1.17.7.1 1.17.7.3]
K02437	gcvH, GCSH; glycine cleavage system H protein
K00605	gcvT, AMT; aminomethyltransferase [EC:2.1.2.10]
K00104	glcD; glycolate oxidase [EC:1.1.3.15]
K11472	glcE; glycolate oxidase FAD binding subunit
K11473	glcF; glycolate oxidase iron-sulfur subunit
K00281	GLDC, gcvP; glycine dehydrogenase [EC:1.4.4.2]
K01091	gph; phosphoglycolate phosphatase [EC:3.1.3.18]
K00018	hprA; glycerate dehydrogenase [EC:1.1.1.29]
K08683	HSD17B10; 3-hydroxyacyl-CoA dehydrogenase / 3-hydroxy-2-methylbutyryl-CoA dehydrogenase [EC:1.1.1.35 1.1.1.178]
K00795	ispA; farnesyl diphosphate synthase [EC:2.5.1.1 2.5.1.10]
K00991	ispD; 2-C-methyl-D-erythritol 4-phosphate cytidylyltransferase [EC:2.7.7.60]
K00919	ispE; 4-diphosphocytidyl-2-C-methyl-D-erythritol kinase [EC:2.7.1.148]
K01770	ispF; 2-C-methyl-D-erythritol 2,4-cyclodiphosphate synthase [EC:4.6.1.12]
K03527	ispH, lytB; 4-hydroxy-3-methylbut-2-en-1-yl diphosphate reductase [EC:1.17.7.4]
K03781	katE, CAT, catB, srpA; catalase [EC:1.11.1.6]
K00024	mdh; malate dehydrogenase [EC:1.1.1.37]
K00641	metX; homoserine O-acetyltransferase/O-succinyltransferase [EC:2.3.1.31 2.3.1.46]
K00940	ndk, NME; nucleoside-diphosphate kinase [EC:2.7.4.6]
K00164	OGDH, sucA; 2-oxoglutarate dehydrogenase E1 component [EC:1.2.4.2]
K01965	PCCA, pccA; propionyl-CoA carboxylase alpha chain [EC:6.4.1.3]
K01966	PCCB, pccB; propionyl-CoA carboxylase beta chain [EC:6.4.1.3 2.1.3.15]

K04782	pchB; isochorismate pyruvate lyase [EC:4.2.99.21]
K01952	PFAS, purL; phosphoribosylformylglycinamidine synthase [EC:6.3.5.3]
K21071	pfk, pfp; ATP-dependent phosphofructokinase / diphosphate-dependent phosphofructokinase [EC:2.7.1.11 2.7.1.90]
K03737	por, nifJ; pyruvate-ferredoxin/flavodoxin oxidoreductase [EC:1.2.7.1 1.2.7.-]
K01923	purC; phosphoribosylaminoimidazole-succinocarboxamide synthase [EC:6.3.2.6]
K01945	purD; phosphoribosylamine---glycine ligase [EC:6.3.4.13]
K01588	purE; 5-(carboxyamino)imidazole ribonucleotide mutase [EC:5.4.99.18]
K00602	purH; phosphoribosylaminoimidazolecarboxamide formyltransferase / IMP cyclohydrolase [EC:2.1.2.3 3.5.4.10]
K01589	purK; 5-(carboxyamino)imidazole ribonucleotide synthase [EC:6.3.4.18]
K01933	purM; phosphoribosylformylglycinamidine cyclo-ligase [EC:6.3.3.1]
K11175	purN; phosphoribosylglycinamide formyltransferase 1 [EC:2.1.2.2]
K08289	purT; phosphoribosylglycinamide formyltransferase 2 [EC:2.1.2.2]
K01601	rbcL; ribulose-bisphosphate carboxylase large chain [EC:4.1.1.39]
K01790	rfbC, rmlC; dTDP-4-dehydrorhamnose 3,5-epimerase [EC:5.1.3.13]
K00067	rfbD, rmlD; dTDP-4-dehydrorhamnose reductase [EC:1.1.1.133]
K00239	sdhA, frdA; succinate dehydrogenase / fumarate reductase, flavoprotein subunit [EC:1.3.5.1 1.3.5.4]
K00240	sdhB, frdB; succinate dehydrogenase / fumarate reductase, iron-sulfur subunit [EC:1.3.5.1 1.3.5.4]
K00241	sdhC, frdC; succinate dehydrogenase / fumarate reductase, cytochrome b subunit
K00242	sdhD, frdD; succinate dehydrogenase / fumarate reductase, membrane anchor subunit
K06013	STE24; STE24 endopeptidase [EC:3.4.24.84]
K01903	sucC; succinyl-CoA synthetase beta subunit [EC:6.2.1.5]
K01902	sucD; succinyl-CoA synthetase alpha subunit [EC:6.2.1.5]
K01638	aceB, glcB; malate synthase [EC:2.3.3.9]
K22225	ahbAB; siroheme decarboxylase [EC:4.1.1.111]
K22226	ahbC; Fe-coproporphyrin III synthase
K00138	aldB; aldehyde dehydrogenase [EC:1.2.1.-]
K04034	bchE; anaerobic magnesium-protoporphyrin IX monomethyl ester cyclase [EC:1.21.98.3]
K17103	CHO1, pssA; CDP-diacylglycerol---serine O-phosphatidyltransferase [EC:2.7.8.8]
K02303	cobA; uroporphyrin-III C-methyltransferase [EC:2.1.1.107]
K06134	COQ7; 3-demethoxyubiquinol 3-hydroxylase [EC:1.14.99.60]
K02257	COX10, ctaB, cyoE; heme o synthase [EC:2.5.1.141]

K02259	COX15, ctaA; cytochrome c oxidase assembly protein subunit 15
K00228	CPOX, hemF; coproporphyrinogen III oxidase [EC:1.3.3.3]
K02291	crtB; 15-cis-phytoene synthase [EC:2.5.1.32]
K00981	E2.7.7.41, CDS1, CDS2, cdsA; phosphatidate cytidylyltransferase [EC:2.7.7.41]
K01637	E4.1.3.1, aceA; isocitrate lyase [EC:4.1.3.1]
K01640	E4.1.3.4, HMGCL, hmgL; hydroxymethylglutaryl-CoA lyase [EC:4.1.3.4]
K00111	glpA, glpD; glycerol-3-phosphate dehydrogenase [EC:1.1.5.3]
K02439	glpE; thiosulfate sulfurtransferase [EC:2.8.1.1]
K00057	gpsA; glycerol-3-phosphate dehydrogenase (NAD(P)+) [EC:1.1.1.94]
K02492	hemA; glutamyl-tRNA reductase [EC:1.2.1.70]
K01698	hemB, ALAD; porphobilinogen synthase [EC:4.2.1.24]
K01749	hemC, HMBS; hydroxymethylbilane synthase [EC:2.5.1.61]
K01719	hemD, UROS; uroporphyrinogen-III synthase [EC:4.2.1.75]
K01599	hemE, UROD; uroporphyrinogen decarboxylase [EC:4.1.1.37]
K01772	hemH, FECH; protoporphyrin/coproporphyrin ferrochelatase [EC:4.99.1.1 4.99.1.9]
K01845	hemL; glutamate-1-semialdehyde 2,1-aminomutase [EC:5.4.3.8]
K02495	hemN, hemZ; oxygen-independent coproporphyrinogen III oxidase [EC:1.3.98.3]
K02496	hemX; uroporphyrin-III C-methyltransferase [EC:2.1.1.107]
K00760	hprT, hpt, HPRT1; hypoxanthine phosphoribosyltransferase [EC:2.4.2.8]
K00088	IMPDH, guaB; IMP dehydrogenase [EC:1.1.1.205]
K02523	ispB; octaprenyl-diphosphate synthase [EC:2.5.1.90]
K03782	katG; catalase-peroxidase [EC:1.11.1.21]
K02548	menA; 1,4-dihydroxy-2-naphthoate polyprenyltransferase [EC:2.5.1.74]
K01661	menB; naphthoate synthase [EC:4.1.3.36]
K02551	menD; 2-succinyl-5-enolpyruvyl-6-hydroxy-3-cyclohexene-1-carboxylate synthase [EC:2.2.1.9]
K01911	menE; O-succinylbenzoic acid---CoA ligase [EC:6.2.1.26]
K00791	miaA, TRIT1; tRNA dimethylallyltransferase [EC:2.5.1.75]
K00606	panB; 3-methyl-2-oxobutanoate hydroxymethyltransferase [EC:2.1.2.11]
K00655	plsC; 1-acyl-sn-glycerol-3-phosphate acyltransferase [EC:2.3.1.51]
K03621	plsX; phosphate acyltransferase [EC:2.3.1.274]
K08591	plsY; acyl phosphate:glycerol-3-phosphate acyltransferase [EC:2.3.1.275]

K09913	ppnP; purine/pyrimidine-nucleoside phosphorylase [EC:2.4.2.1 2.4.2.2]
K01613	psd, PISD; phosphatidylserine decarboxylase [EC:4.1.1.65]
K14652	ribBA; 3,4-dihydroxy 2-butanone 4-phosphate synthase / GTP cyclohydrolase II [EC:4.1.99.12 3.5.4.25]
K11752	ribD; diaminohydroxyphosphoribosylaminopyrimidine deaminase / 5-amino-6-(5-phosphoribosylamino)uracil reductase [EC:3.5.4.26 1.1.1.193]
K00793	ribE, RIB5; riboflavin synthase [EC:2.5.1.9]
K11753	ribF; riboflavin kinase / FMN adenylyltransferase [EC:2.7.1.26 2.7.7.2]
K00794	ribH, RIB4; 6,7-dimethyl-8-ribityllumazine synthase [EC:2.5.1.78]
K03787	surE; 5'-nucleotidase [EC:3.1.3.5]
K03179	ubiA; 4-hydroxybenzoate polyprenyltransferase [EC:2.5.1.39]
K03181	ubiC; chorismate--pyruvate lyase [EC:4.1.3.40]
K03182	ubiD; 4-hydroxy-3-polyprenylbenzoate decarboxylase [EC:4.1.1.98]
K03183	ubiE; demethylmenaquinone methyltransferase / 2-methoxy-6-polyprenyl-1,4-benzoquinol methylase [EC:2.1.1.163 2.1.1.201]
K00568	ubiG; 2-polyprenyl-6-hydroxyphenyl methylase / 3-demethylubiquinone-9 3-methyltransferase [EC:2.1.1.222 2.1.1.64]
K00806	uppS; undecaprenyl diphosphate synthase [EC:2.5.1.31]
K03809	wrbA; NAD(P)H dehydrogenase (quinone) [EC:1.6.5.2]
K10804	tesA; acyl-CoA thioesterase I [EC:3.1.2.- 3.1.2.2 3.1.1.2 3.1.1.5]
K10806	yciA; acyl-CoA thioesterase YciA [EC:3.1.2.-]
K00833	bioA; adenosylmethionine---8-amino-7-oxononanoate aminotransferase [EC:2.6.1.62]
K01012	bioB; biotin synthase [EC:2.8.1.6]
K02169	bioC; malonyl-CoA O-methyltransferase [EC:2.1.1.197]
K01935	bioD; dethiobiotin synthetase [EC:6.3.3.3]
K00652	bioF; 8-amino-7-oxononanoate synthase [EC:2.3.1.47]
K02170	bioH; pimeloyl-[acyl-carrier protein] methyl ester esterase [EC:3.1.1.85]
K03524	birA; BirA family transcriptional regulator, biotin operon repressor / biotin---[acetyl-CoA-carboxylase] ligase [EC:6.3.4.15]
K00647	fabB; 3-oxoacyl-[acyl-carrier-protein] synthase I [EC:2.3.1.41]
K09458	fabF, OXSM, CEM1; 3-oxoacyl-[acyl-carrier-protein] synthase II [EC:2.3.1.179]
K02372	fabZ; 3-hydroxyacyl-[acyl-carrier-protein] dehydratase [EC:4.2.1.59]
K01907	AACS, acsA; acetoacetyl-CoA synthetase [EC:6.2.1.16]
K17865	croR; 3-hydroxybutyryl-CoA dehydratase [EC:4.2.1.55]
K01039	gctA; glutaconate CoA-transferase, subunit A [EC:2.8.3.12]

K01040	gctB; glutaconate CoA-transferase, subunit B [EC:2.8.3.12]
K00023	phbB; acetoacetyl-CoA reductase [EC:1.1.1.36]
K03821	phbC, phaC; polyhydroxyalkanoate synthase subunit PhaC [EC:2.3.1.-]
K00634	ptb; phosphate butyryltransferase [EC:2.3.1.19]
K14731	mlhB, chnC; epsilon-lactone hydrolase [EC:3.1.1.83]
K01595	ppc; phosphoenolpyruvate carboxylase [EC:4.1.1.31]
K00855	PRK, prkB; phosphoribulokinase [EC:2.7.1.19]
K00925	ackA; acetate kinase [EC:2.7.2.1]
K01491	folD; methylenetetrahydrofolate dehydrogenase (NADP+) / methenyltetrahydrofolate cyclohydrolase [EC:1.5.1.5 3.5.4.9]
K09709	meh; 3-methylfumaryl-CoA hydratase [EC:4.2.1.153]
K00297	metF, MTHFR; methylenetetrahydrofolate reductase (NADPH) [EC:1.5.1.20]
K01847	MUT; methylmalonyl-CoA mutase [EC:5.4.99.2]
K01007	pps, ppsA; pyruvate, water dikinase [EC:2.7.9.2]
K00124	fdoH, fdsB; formate dehydrogenase iron-sulfur subunit
K00127	fdoI, fdsG; formate dehydrogenase subunit gamma
K00027	ME2, sfcA, maeA; malate dehydrogenase (oxaloacetate-decarboxylating) [EC:1.1.1.38]
K00413	CYC1, CYT1, petC; ubiquinol-cytochrome c reductase cytochrome c1 subunit
K00412	CYTB, petB; ubiquinol-cytochrome c reductase cytochrome b subunit
K00411	UQCRFS1, RIP1, petA; ubiquinol-cytochrome c reductase iron-sulfur subunit [EC:7.1.1.8]
K01358	clpP, CLPP; ATP-dependent Clp protease, protease subunit [EC:3.4.21.92]
K03544	clpX, CLPX; ATP-dependent Clp protease ATP-binding subunit ClpX
K13590	dgcB; diguanylate cyclase [EC:2.7.7.65]
K02313	dnaA; chromosomal replication initiator protein
K02314	dnaB; replicative DNA helicase [EC:3.6.4.12]
K03590	ftsA; cell division protein FtsA
K03589	ftsQ; cell division protein FtsQ
K03588	ftsW, spoVE; cell division protein FtsW
K03531	ftsZ; cell division protein FtsZ
K06985	K06985; aspartyl protease family protein
K01338	lon; ATP-dependent Lon protease [EC:3.4.21.53]
K02563	murG; UDP-N-acetylglucosamine--N-acetylmuramyl-(pentapeptide) pyrophosphoryl-undecaprenol N-acetylglucosamine

	transferase [EC:2.4.1.227]
K02488	pleD; two-component system, cell cycle response regulator [EC:2.7.7.65]
K11749	rseP; regulator of sigma E protease [EC:3.4.24.-]
K02586	nifD; nitrogenase molybdenum-iron protein alpha chain [EC:1.18.6.1]
K02588	nifH; nitrogenase iron protein NifH
K02591	nifK; nitrogenase molybdenum-iron protein beta chain [EC:1.18.6.1]
K01061	E3.1.1.45; carboxymethylenebutenolidase [EC:3.1.1.45]
K16844	comC; (2R)-3-sulfolactate dehydrogenase (NADP+) [EC:1.1.1.338]
K01251	E3.3.1.1, ahcY; adenosylhomocysteinase [EC:3.3.1.1]
K01920	gshB; glutathione synthase [EC:6.3.2.3]
K01740	metY; O-acetylhomoserine (thiol)-lyase [EC:2.5.1.49]
K10764	metZ; O-succinylhomoserine sulfhydrylase [EC:2.5.1.-]
K08963	mtnA; methylthioribose-1-phosphate isomerase [EC:5.3.1.23]
K16843	slcC; (S)-sulfolactate dehydrogenase [EC:1.1.1.310]
K16845	suyA; (2R)-sulfolactate sulfo-lyase subunit alpha [EC:4.4.1.24]
K16846	suyB; (2R)-sulfolactate sulfo-lyase subunit beta [EC:4.4.1.24]
K01011	TST, MPST, sseA; thiosulfate/3-mercaptopyruvate sulfurtransferase [EC:2.8.1.1 2.8.1.2]
K01775	alr; alanine racemase [EC:5.1.1.1]
K01921	ddl; D-alanine-D-alanine ligase [EC:6.3.2.4]
K00455	hpaD, hpcB; 3,4-dihydroxyphenylacetate 2,3-dioxygenase [EC:1.13.11.15]
K00151	hpaE, hpcC; 5-carboxymethyl-2-hydroxymuconic-semialdehyde dehydrogenase [EC:1.2.1.60]
K01826	hpaF, hpcD; 5-carboxymethyl-2-hydroxymuconate isomerase [EC:5.3.3.10]
K05921	hpaG; 5-oxopent-3-ene-1,2,5-tricarboxylate decarboxylase / 2-hydroxyhepta-2,4-diene-1,7-dioate isomerase [EC:4.1.1.68 5.3.3.-]
K01924	murC; UDP-N-acetylmuramate--alanine ligase [EC:6.3.2.8]
K01925	murD; UDP-N-acetylmuramoylalanine--D-glutamate ligase [EC:6.3.2.9]
K01776	murI; glutamate racemase [EC:5.1.1.3]
K02337	dnaE; DNA polymerase III subunit alpha [EC:2.7.7.7]
K02316	dnaG; DNA primase [EC:2.7.7.101]
K02338	dnaN; DNA polymerase III subunit beta [EC:2.7.7.7]
K02342	dnaQ; DNA polymerase III subunit epsilon [EC:2.7.7.7]
K02343	dnaX; DNA polymerase III subunit gamma/tau [EC:2.7.7.7]

K02340	holA; DNA polymerase III subunit delta [EC:2.7.7.7]
K02341	holB; DNA polymerase III subunit delta' [EC:2.7.7.7]
K02339	holC; DNA polymerase III subunit chi [EC:2.7.7.7]
K03469	rnhA, RNASEH1; ribonuclease HI [EC:3.1.26.4]
K03470	rnhB; ribonuclease HII [EC:3.1.26.4]
K03111	ssb; single-strand DNA-binding protein
K00799	GST, gst; glutathione S-transferase [EC:2.5.1.18]
K01520	dut, DUT; dUTP pyrophosphatase [EC:3.6.1.23]
K01951	guaA, GMPS; GMP synthase (glutamine-hydrolysing) [EC:6.3.5.2]
K00648	fabH; 3-oxoacyl-[acyl-carrier-protein] synthase III [EC:2.3.1.180]
K05939	aas; acyl-[acyl-carrier-protein]-phospholipid O-acyltransferase / long-chain-fatty-acid--[acyl-carrier-protein] ligase [EC:2.3.1.40 6.2.1.20]
K06445	fadE; acyl-CoA dehydrogenase [EC:1.3.99.-]
K02386	flgA; flagella basal body P-ring formation protein FlgA
K02387	flgB; flagellar basal-body rod protein FlgB
K02388	flgC; flagellar basal-body rod protein FlgC
K02389	flgD; flagellar basal-body rod modification protein FlgD
K02390	flgE; flagellar hook protein FlgE
K02391	flgF; flagellar basal-body rod protein FlgF
K02392	flgG; flagellar basal-body rod protein FlgG
K02393	flgH; flagellar L-ring protein precursor FlgH
K02394	flgI; flagellar P-ring protein precursor FlgI
K02396	flgK; flagellar hook-associated protein 1 FlgK
K02397	flgL; flagellar hook-associated protein 3 FlgL
K02399	flgN; flagella synthesis protein FlgN
K02400	flhA; flagellar biosynthesis protein FlhA
K02401	flhB; flagellar biosynthetic protein FlhB
K02406	fliC; flagellin
K02407	fliD; flagellar hook-associated protein 2
K02408	fliE; flagellar hook-basal body complex protein FliE
K02409	fliF; flagellar M-ring protein FliF

K02411	fliH; flagellar assembly protein FliH
K02412	fliI; flagellum-specific ATP synthase [EC:7.4.2.8]
K02413	fliJ; flagellar FliJ protein
K02414	fliK; flagellar hook-length control protein FliK
K02418	fliOZ, fliO; flagellar protein FliO/FliZ
K02419	fliP; flagellar biosynthetic protein FliP
K02420	fliQ; flagellar biosynthetic protein FliQ
K02421	fliR; flagellar biosynthetic protein FliR
K02422	fliS; flagellar protein FliS
K00287	DHFR, folA; dihydrofolate reductase [EC:1.5.1.3]
K01633	folB; 7,8-dihydroneopterin aldolase/epimerase/oxygenase [EC:4.1.2.25 5.1.99.8 1.13.11.81]
K11754	folC; dihydrofolate synthase / folylpolyglutamate synthase [EC:6.3.2.12 6.3.2.17]
K09007	folE2; GTP cyclohydrolase IB [EC:3.5.4.16]
K00950	folK; 2-amino-4-hydroxy-6-hydroxymethyldihydropteridine diphosphokinase [EC:2.7.6.3]
K00796	folP; dihydropteroate synthase [EC:2.5.1.15]
K03639	moaA, CNX2; GTP 3',8-cyclase [EC:4.1.99.22]
K03637	moaC, CNX3; cyclic pyranopterin monophosphate synthase [EC:4.6.1.17]
K03752	mobA; molybdenum cofactor guanylyltransferase [EC:2.7.7.77]
K03635	MOCS2B, moaE; molybdopterin synthase catalytic subunit [EC:2.8.1.12]
K03750	moeA; molybdopterin molybdotransferase [EC:2.10.1.1]
K03831	mogA; molybdopterin adenylyltransferase [EC:2.7.7.75]
K08310	nudB, ntpA; dihydroneopterin triphosphate diphosphatase [EC:3.6.1.67]
K03342	pabBC; para-aminobenzoate synthetase / 4-amino-4-deoxychorismate lyase [EC:2.6.1.85 4.1.3.38]
K01724	PCBD, phhB; 4a-hydroxytetrahydrobiopterin dehydratase [EC:4.2.1.96]
K01113	phoD; alkaline phosphatase D [EC:3.1.3.1]
K06920	queC; 7-cyano-7-deazaguanine synthase [EC:6.3.4.20]
K01737	queD, ptpS, PTS; 6-pyruvoyltetrahydropterin/6-carboxytetrahydropterin synthase [EC:4.2.3.12 4.1.2.50]
K10026	queE; 7-carboxy-7-deazaguanine synthase [EC:4.3.99.3]
K06879	queF; 7-cyano-7-deazaguanine reductase [EC:1.7.1.13]
K04564	SOD2; superoxide dismutase, Fe-Mn family [EC:1.15.1.1]
K01628	fucA; L-fuculose-phosphate aldolase [EC:4.1.2.17]

K01255	CARP, pepA; leucyl aminopeptidase [EC:3.4.11.1]
K06048	gshA, ybdK; glutamate---cysteine ligase / carboxylate-amine ligase [EC:6.3.2.2 6.3.-.-]
K01270	pepD; dipeptidase D [EC:3.4.13.-]
K01256	pepN; aminopeptidase N [EC:3.4.11.2]
K07160	pxpA; 5-oxoprolinase (ATP-hydrolysing) subunit A [EC:3.5.2.9]
K00864	glpK, GK; glycerol kinase [EC:2.7.1.30]
K01046	lip, TGL2; triacylglycerol lipase [EC:3.1.1.3]
K19002	mgs, bgsB; 1,2-diacylglycerol 3-alpha-glucosyltransferase [EC:2.4.1.337]
K13622	btaA; S-adenosylmethionine-diacylglycerol 3-amino-3-carboxypropyl transferase
K13623	btaB; S-adenosylmethionine-diacylgycerolhomoserine-N-methlytransferase
K06131	clsA_B; cardiolipin synthase A/B [EC:2.7.8.-]
K01126	E3.1.4.46, glpQ, ugpQ; glycerophosphoryl diester phosphodiesterase [EC:3.1.4.46]
K01095	pgpA; phosphatidylglycerophosphatase A [EC:3.1.3.27]
K00995	pgsA, PGS1; CDP-diacylglycerol---glycerol-3-phosphate 3-phosphatidyltransferase [EC:2.7.8.5]
K00108	betA, CHDH; choline dehydrogenase [EC:1.1.99.1]
K00499	CMO; choline monooxygenase [EC:1.14.15.7]
K00042	garR, glxR; 2-hydroxy-3-oxopropionate reductase [EC:1.1.1.60]
K01816	hyi, gip; hydroxypyruvate isomerase [EC:5.3.1.22]
K01433	purU; formyltetrahydrofolate deformylase [EC:3.5.1.10]
K04066	priA; primosomal protein N' (replication factor Y) (superfamily II helicase) [EC:3.6.4.-]
K03553	recA; recombination protein RecA
K03655	recG; ATP-dependent DNA helicase RecG [EC:3.6.4.12]
K03584	recO; DNA repair protein RecO (recombination protein O)
K06187	recR; recombination protein RecR
K03550	ruvA; holliday junction DNA helicase RuvA [EC:3.6.4.12]
K03551	ruvB; holliday junction DNA helicase RuvB [EC:3.6.4.12]
K01159	ruvC; crossover junction endodeoxyribonuclease RuvC [EC:3.1.22.4]
K03644	lipA, LIAS, LIP1, LIP5; lipoyl synthase [EC:2.8.1.8]
K03801	lipB; lipoyl(octanoyl) transferase [EC:2.3.1.181]
K07264	arnT, pmrK; 4-amino-4-deoxy-L-arabinose transferase [EC:2.4.2.43]
K03760	eptA, pmrC; lipid A ethanolaminephosphotransferase [EC:2.7.8.43]

K03271	gmhA, lpcA; D-sedoheptulose 7-phosphate isomerase [EC:5.3.1.28]
K03273	gmhB; D-glycero-D-manno-heptose 1,7-bisphosphate phosphatase [EC:3.1.3.82 3.1.3.83]
K03274	gmhD, rfaD; ADP-L-glycero-D-manno-heptose 6-epimerase [EC:5.1.3.20]
K01627	kdsA; 2-dehydro-3-deoxyphosphooctonate aldolase (KDO 8-P synthase) [EC:2.5.1.55]
K00979	kdsB; 3-deoxy-manno-octulosonate cytidylyltransferase (CMP-KDO synthetase) [EC:2.7.7.38]
K03270	kdsC; 3-deoxy-D-manno-octulosonate 8-phosphate phosphatase (KDO 8-P phosphatase) [EC:3.1.3.45]
K06041	kdsD, kpsF; arabinose-5-phosphate isomerase [EC:5.3.1.13]
K02527	kdtA, waaA; 3-deoxy-D-manno-octulosonic-acid transferase [EC:2.4.99.12 2.4.99.13 2.4.99.14 2.4.99.15]
K00677	lpxA; UDP-N-acetylglucosamine acyltransferase [EC:2.3.1.129]
K00748	lpxB; lipid-A-disaccharide synthase [EC:2.4.1.182]
K02535	lpxC; UDP-3-O-[3-hydroxymyristoyl] N-acetylglucosamine deacetylase [EC:3.5.1.108]
K02536	lpxD; UDP-3-O-[3-hydroxymyristoyl] glucosamine N-acyltransferase [EC:2.3.1.191]
K03269	lpxH; UDP-2,3-diacylglucosamine hydrolase [EC:3.6.1.54]
K00912	lpxK; tetraacyldisaccharide 4'-kinase [EC:2.7.1.130]
K02517	lpxL, htrB; Kdo2-lipid IVA lauroyltransferase/acyltransferase [EC:2.3.1.241 2.3.1.-]
K21344	rfaE1; D-glycero-beta-D-manno-heptose-7-phosphate kinase [EC:2.7.1.167]
K21345	rfaE2; D-glycero-beta-D-manno-heptose 1-phosphate adenylyltransferase [EC:2.7.7.70]
K02841	waaC, rfaC; heptosyltransferase I [EC:2.4.-.-]
K02843	waaF, rfaF; heptosyltransferase II [EC:2.4.-.-]
K02844	waaG, rfaG; UDP-glucose:(heptosyl)LPS alpha-1,3-glucosyltransferase [EC:2.4.1.-]
K02849	waaQ, rfaQ; heptosyltransferase III [EC:2.4.-.-]
K03695	clpB; ATP-dependent Clp protease ATP-binding subunit ClpB
K04565	SOD1; superoxide dismutase, Cu-Zn family [EC:1.15.1.1]
K04043	dnaK, HSPA9; molecular chaperone DnaK
K04077	groEL, HSPD1; chaperonin GroEL
K01928	murE; UDP-N-acetylmuramoyl-L-alanyl-D-glutamate--2,6-diaminopimelate ligase [EC:6.3.2.13]
K01929	murF; UDP-N-acetylmuramoyl-tripeptide--D-alanyl-D-alanine ligase [EC:6.3.2.10]
K18014	kal; 3-aminobutyryl-CoA ammonia-lyase [EC:4.3.1.14]
K01843	kamA; lysine 2,3-aminomutase [EC:5.4.3.2]
K18011	kamE; beta-lysine 5,6-aminomutase beta subunit [EC:5.4.3.3]
K18013	kce; 3-keto-5-aminohexanoate cleavage enzyme [EC:2.3.1.247]

K18012	kdd; L-erythro-3,5-diaminohexanoate dehydrogenase [EC:1.4.1.11]
K17686	copA, ctpA, ATP7; P-type Cu+ transporter [EC:7.2.2.8]
K01908	ACSS3, prpE; propionyl-CoA synthetase [EC:6.2.1.17]
K19745	acuI; acrylyl-CoA reductase (NADPH) [EC:1.3.1.-]
K01525	apaH; bis(5'-nucleosyl)-tetraphosphatase (symmetrical) [EC:3.6.1.41]
K00759	APRT, apt; adenine phosphoribosyltransferase [EC:2.4.2.7]
K02108	ATPF0A, atpB; F-type H+-transporting ATPase subunit a
K02109	ATPF0B, atpF; F-type H+-transporting ATPase subunit b
K02110	ATPF0C, atpE; F-type H+-transporting ATPase subunit c
K02111	ATPF1A, atpA; F-type H+/Na+-transporting ATPase subunit alpha [EC:7.1.2.2 7.2.2.1]
K02112	ATPF1B, atpD; F-type H+/Na+-transporting ATPase subunit beta [EC:7.1.2.2 7.2.2.1]
K02113	ATPF1D, atpH; F-type H+-transporting ATPase subunit delta
K02114	ATPF1E, atpC; F-type H+-transporting ATPase subunit epsilon
K02115	ATPF1G, atpG; F-type H+-transporting ATPase subunit gamma
K04719	bluB; 5,6-dimethylbenzimidazole synthase [EC:1.13.11.79]
K01674	cah; carbonic anhydrase [EC:4.2.1.1]
K02227	cbiB, cobD; adenosylcobinamide-phosphate synthase [EC:6.3.1.10]
K02188	cbiD; cobalt-precorrin-5B (C1)-methyltransferase [EC:2.1.1.195]
K02189	cbiG; cobalt-precorrin 5A hydrolase [EC:3.7.1.12]
K03795	cbiX; sirohydrochlorin cobaltochelatase [EC:4.99.1.3]
K00404	ccoN; cytochrome c oxidase cbb3-type subunit I [EC:7.1.1.9]
K00405	ccoO; cytochrome c oxidase cbb3-type subunit II
K00406	ccoP; cytochrome c oxidase cbb3-type subunit III
K00407	ccoQ; cytochrome c oxidase cbb3-type subunit IV
K13038	coaBC, dfp; phosphopantothenoylcysteine decarboxylase / phosphopantothenate---cysteine ligase [EC:4.1.1.36 6.3.2.5]
K00859	coaE; dephospho-CoA kinase [EC:2.7.1.24]
K03525	coaX; type III pantothenate kinase [EC:2.7.1.33]
K19221	cobA, btuR; cob(I)alamin adenosyltransferase [EC:2.5.1.17]
K02224	cobB-cbiA; cobyrinic acid a,c-diamide synthase [EC:6.3.5.9 6.3.5.11]
K02226	cobC, phpB; alpha-ribazole phosphatase [EC:3.1.3.73]
K02225	cobC1, cobC; cobalamin biosynthetic protein CobC

K06042	cobH-cbiC; precorrin-8X/cobalt-precorrin-8 methylmutase [EC:5.4.99.61 5.4.99.60]
K03394	cobI-cbiL; precorrin-2/cobalt-factor-2 C20-methyltransferase [EC:2.1.1.130 2.1.1.151]
K00595	cobL; precorrin-6Y C5,15-methyltransferase (decarboxylating) [EC:2.1.1.132]
K05936	cobM, cbiF; precorrin-4/cobalt-precorrin-4 C11-methyltransferase [EC:2.1.1.133 2.1.1.271]
K02231	cobP, cobU; adenosylcobinamide kinase / adenosylcobinamide-phosphate guanylyltransferase [EC:2.7.1.156 2.7.7.62]
K02232	cobQ, cbiP; adenosylcobyric acid synthase [EC:6.3.5.10]
K05979	comB; 2-phosphosulfolactate phosphatase [EC:3.1.3.71]
K02258	COX11, ctaG; cytochrome c oxidase assembly protein subunit 11
K02274	coxA, ctaD; cytochrome c oxidase subunit I [EC:7.1.1.9]
K02275	coxB, ctaC; cytochrome c oxidase subunit II [EC:7.1.1.9]
K02276	coxC, ctaE; cytochrome c oxidase subunit III [EC:7.1.1.9]
K01725	cynS; cyanate lyase [EC:4.2.1.104]
K01673	cynT, can; carbonic anhydrase [EC:4.2.1.1]
K00390	cysH; phosphoadenosine phosphosulfate reductase [EC:1.8.4.8 1.8.4.10]
K00381	cysI; sulfite reductase (NADPH) hemoprotein beta-component [EC:1.8.1.2]
K07258	dacC, dacA, dacD; serine-type D-Ala-D-Ala carboxypeptidase (penicillin-binding protein 5/6) [EC:3.4.16.4]
K00285	dadA; D-amino-acid dehydrogenase [EC:1.4.5.1]
K01494	dcd; dCTP deaminase [EC:3.5.4.13]
K01129	dgt; dGTPase [EC:3.1.5.1]
K00254	DHODH, pyrD; dihydroorotate dehydrogenase [EC:1.3.5.2]
K20035	dmdC; 3-(methylthio)propanoyl-CoA dehydrogenase [EC:1.3.8.-]
K07306	dmsA; anaerobic dimethyl sulfoxide reductase subunit A [EC:1.8.5.3]
K07307	dmsB; anaerobic dimethyl sulfoxide reductase subunit B
K00525	E1.17.4.1A, nrdA, nrdE; ribonucleoside-diphosphate reductase alpha chain [EC:1.17.4.1]
K00526	E1.17.4.1B, nrdB, nrdF; ribonucleoside-diphosphate reductase beta chain [EC:1.17.4.1]
K05301	E1.8.2.1; sulfite dehydrogenase [EC:1.8.2.1]
K05934	E2.1.1.131, cobJ, cbiH; precorrin-3B C17-methyltransferase [EC:2.1.1.131]
K00768	E2.4.2.21, cobU, cobT; nicotinate-nucleotide--dimethylbenzimidazole phosphoribosyltransferase [EC:2.4.2.21]
K00856	E2.7.1.20, ADK; adenosine kinase [EC:2.7.1.20]
K00942	E2.7.4.8, gmk; guanylate kinase [EC:2.7.4.8]
K00954	E2.7.7.3A, coaD, kdtB; pantetheine-phosphate adenylyltransferase [EC:2.7.7.3]

K02233	E2.7.8.26, cobS, cobV; adenosylcobinamide-GDP ribazoletransferase [EC:2.7.8.26]
K03426	E3.6.1.22, NUDT12, nudC; NAD+ diphosphatase [EC:3.6.1.22]
K01796	E5.1.99.4, AMACR, mcr; alpha-methylacyl-CoA racemase [EC:5.1.99.4]
K01950	E6.3.5.1, NADSYN1, QNS1, nadE; NAD+ synthase (glutamine-hydrolysing) [EC:6.3.5.1]
K01968	E6.4.1.4A; 3-methylcrotonyl-CoA carboxylase alpha subunit [EC:6.4.1.4]
K01969	E6.4.1.4B; 3-methylcrotonyl-CoA carboxylase beta subunit [EC:6.4.1.4]
K03472	epd; D-erythrose 4-phosphate dehydrogenase [EC:1.2.1.72]
K17230	fccA; cytochrome subunit of sulfide dehydrogenase
K17229	fccB; sulfide dehydrogenase [flavocytochrome c] flavoprotein chain [EC:1.8.2.3]
K01759	GLO1, gloA; lactoylglutathione lyase [EC:4.4.1.5]
K01069	gloB, gloC, HAGH; hydroxyacylglutathione hydrolase [EC:3.1.2.6]
K01451	hipO; hippurate hydrolase [EC:3.5.1.32]
K02509	hpaH; 2-oxo-hept-3-ene-1,7-dioate hydratase [EC:4.2.1.-]
K02510	hpaI, hpcH; 4-hydroxy-2-oxoheptanedioate aldolase [EC:4.1.2.52]
K04487	iscS, NFS1; cysteine desulfurase [EC:2.8.1.7]
K00253	IVD, ivd; isovaleryl-CoA dehydrogenase [EC:1.3.8.4]
K00102	LDHD, dld; D-lactate dehydrogenase (cytochrome) [EC:1.1.2.4]
K13766	liuC; methylglutaconyl-CoA hydratase [EC:4.2.1.18]
K15228	mauA; methylamine dehydrogenase light chain [EC:1.4.9.1]
K15229	mauB; methylamine dehydrogenase heavy chain [EC:1.4.9.1]
K00798	MMAB, pduO; cob(I)alamin adenosyltransferase [EC:2.5.1.17]
K00020	mmsB, HIBADH; 3-hydroxyisobutyrate dehydrogenase [EC:1.1.1.31]
K01000	mraY; phospho-N-acetylmuramoyl-pentapeptide-transferase [EC:2.7.8.13]
K05366	mrcA; penicillin-binding protein 1A [EC:2.4.1.129 3.4.16.4]
K01934	MTHFS; 5-formyltetrahydrofolate cyclo-ligase [EC:6.3.3.2]
K03517	nadA; quinolinate synthase [EC:2.5.1.72]
K00767	nadC, QPRT; nicotinate-nucleotide pyrophosphorylase (carboxylating) [EC:2.4.2.19]
K00969	nadD; nicotinate-nucleotide adenylyltransferase [EC:2.7.7.18]
K16165	nagK; fumarylpyruvate hydrolase [EC:3.7.1.20]
K02567	napA; nitrate reductase (cytochrome) [EC:1.9.6.1]
K02568	napB; nitrate reductase (cytochrome), electron transfer subunit

K00372	nasA; assimilatory nitrate reductase catalytic subunit [EC:1.7.99.-]
K00459	ncd2, npd; nitronate monooxygenase [EC:1.13.12.16]
K03953	NDUFA9; NADH dehydrogenase (ubiquinone) 1 alpha subcomplex subunit 9
K00362	nirB; nitrite reductase (NADH) large subunit [EC:1.7.1.15]
K00363	nirD; nitrite reductase (NADH) small subunit [EC:1.7.1.15]
K15864	nirS; nitrite reductase (NO-forming) / hydroxylamine reductase [EC:1.7.2.1 1.7.99.1]
K04561	norB; nitric oxide reductase subunit B [EC:1.7.2.5]
K02305	norC; nitric oxide reductase subunit C
K00376	nosZ; nitrous-oxide reductase [EC:1.7.2.4]
K21636	nrdD; ribonucleoside-triphosphate reductase (formate) [EC:1.1.98.6]
K01515	nudF; ADP-ribose pyrophosphatase [EC:3.6.1.13]
K18453	NUDT23; ADP-ribose/FAD diphosphatase [EC:3.6.1.13 3.6.1.18]
K00330	nuoA; NADH-quinone oxidoreductase subunit A [EC:7.1.1.2]
K00331	nuoB; NADH-quinone oxidoreductase subunit B [EC:7.1.1.2]
K00332	nuoC; NADH-quinone oxidoreductase subunit C [EC:7.1.1.2]
K00333	nuoD; NADH-quinone oxidoreductase subunit D [EC:7.1.1.2]
K00334	nuoE; NADH-quinone oxidoreductase subunit E [EC:7.1.1.2]
K00335	nuoF; NADH-quinone oxidoreductase subunit F [EC:7.1.1.2]
K00336	nuoG; NADH-quinone oxidoreductase subunit G [EC:7.1.1.2]
K00337	nuoH; NADH-quinone oxidoreductase subunit H [EC:7.1.1.2]
K00338	nuoI; NADH-quinone oxidoreductase subunit I [EC:7.1.1.2]
K00339	nuoJ; NADH-quinone oxidoreductase subunit J [EC:7.1.1.2]
K00340	nuoK; NADH-quinone oxidoreductase subunit K [EC:7.1.1.2]
K00341	nuoL; NADH-quinone oxidoreductase subunit L [EC:7.1.1.2]
K00342	nuoM; NADH-quinone oxidoreductase subunit M [EC:7.1.1.2]
K00343	nuoN; NADH-quinone oxidoreductase subunit N [EC:7.1.1.2]
K02609	paaA; ring-1,2-phenylacetyl-CoA epoxidase subunit PaaA [EC:1.14.13.149]
K02610	paaB; ring-1,2-phenylacetyl-CoA epoxidase subunit PaaB
K02611	paaC; ring-1,2-phenylacetyl-CoA epoxidase subunit PaaC [EC:1.14.13.149]
K02612	paaD; ring-1,2-phenylacetyl-CoA epoxidase subunit PaaD
K02613	paaE; ring-1,2-phenylacetyl-CoA epoxidase subunit PaaE

K15866	paaG; 2-(1,2-epoxy-1,2-dihydrophenyl)acetyl-CoA isomerase [EC:5.3.3.18]
K02614	paaI; acyl-CoA thioesterase [EC:3.1.2.-]
K01026	pct; propionate CoA-transferase [EC:2.8.3.1]
K00097	pdxA; 4-hydroxythreonine-4-phosphate dehydrogenase [EC:1.1.1.262]
K03474	pdxJ; pyridoxine 5-phosphate synthase [EC:2.6.99.2]
K03430	phnW; 2-aminoethylphosphonate-pyruvate transaminase [EC:2.6.1.37]
K05306	phnX; phosphonoacetaldehyde hydrolase [EC:3.11.1.1]
K00324	pntA; H+-translocating NAD(P) transhydrogenase subunit alpha [EC:1.6.1.2 7.1.1.1]
K00325	pntB; H+-translocating NAD(P) transhydrogenase subunit beta [EC:1.6.1.2 7.1.1.1]
K00858	ppnK, NADK; NAD+ kinase [EC:2.7.1.23]
K01524	ppx-gppA; exopolyphosphatase / guanosine-5'-triphosphate,3'-diphosphate pyrophosphatase [EC:3.6.1.11 3.6.1.40]
K00762	pyrE; orotate phosphoribosyltransferase [EC:2.4.2.10]
K01591	pyrF; orotidine-5'-phosphate decarboxylase [EC:4.1.1.23]
K01937	pyrG, CTPS; CTP synthase [EC:6.3.4.2]
K09903	pyrH; uridylate kinase [EC:2.7.4.22]
K02825	pyrR; pyrimidine operon attenuation protein / uracil phosphoribosyltransferase [EC:2.4.2.9]
K02428	rdgB; XTP/dITP diphosphohydrolase [EC:3.6.1.66]
K00951	relA; GTP pyrophosphokinase [EC:2.7.6.5]
K06949	rsgA, engC; ribosome biogenesis GTPase / thiamine phosphate phosphatase [EC:3.6.1.- 3.1.3.100]
K01008	selD, SEPHS; selenide, water dikinase [EC:2.7.9.3]
K17222	soxA; L-cysteine S-thiosulfotransferase [EC:2.8.5.2]
K17224	soxB; S-sulfosulfanyl-L-cysteine sulfohydrolase [EC:3.1.6.20]
K17225	soxC; sulfane dehydrogenase subunit SoxC
K22622	soxD; S-disulfanyl-L-cysteine oxidoreductase SoxD [EC:1.8.2.6]
K17223	soxX; L-cysteine S-thiosulfotransferase [EC:2.8.5.2]
K17226	soxY; sulfur-oxidizing protein SoxY
K17227	soxZ; sulfur-oxidizing protein SoxZ
K01139	spoT; GTP diphosphokinase / guanosine-3',5'-bis(diphosphate) 3'-diphosphatase [EC:2.7.6.5 3.1.7.2]
K03147	thiC; phosphomethylpyrimidine synthase [EC:4.1.99.17]
K00788	thiE; thiamine-phosphate pyrophosphorylase [EC:2.5.1.3]
K03149	thiG; thiazole synthase [EC:2.8.1.10]

K00946	thiL; thiamine-monophosphate kinase [EC:2.7.4.16]
K00560	thyA, TYMS; thymidylate synthase [EC:2.1.1.45]
K00943	tmk, DTYMK; dTMP kinase [EC:2.7.4.9]
K00761	upp, UPRT; uracil phosphoribosyltransferase [EC:2.4.2.9]
K01465	URA4, pyrC; dihydroorotase [EC:3.5.2.3]
K03852	xsc; sulfoacetaldehyde acetyltransferase [EC:2.3.3.15]
K23548	hupU; uptake hydrogenase small subunit [EC:1.12.99.6]
K23549	hupV; uptake hydrogenase large subunit [EC:1.12.99.6]
K06282	hyaA, hybO; hydrogenase small subunit [EC:1.12.99.6]
K06281	hyaB, hybC; hydrogenase large subunit [EC:1.12.99.6]
K10680	nemA; N-ethylmaleimide reductase [EC:1.-.-.-]
K10679	nfnB, nfsB; nitroreductase / dihydropteridine reductase [EC:1.-.-.- 1.5.1.34]
K06223	dam; DNA adenine methylase [EC:2.1.1.72]
K03572	mutL; DNA mismatch repair protein MutL
K03555	mutS; DNA mismatch repair protein MutS
K03657	uvrD, pcrA; DNA helicase II / ATP-dependent DNA helicase PcrA [EC:3.6.4.12]
K03601	xseA; exodeoxyribonuclease VII large subunit [EC:3.1.11.6]
K03602	xseB; exodeoxyribonuclease VII small subunit [EC:3.1.11.6]
K03743	pncC; nicotinamide-nucleotide amidase [EC:3.5.1.42]
K02575	NRT, narK, nrtP, nasA; MFS transporter, NNP family, nitrate/nitrite transporter
K03671	trxA; thioredoxin 1
K03723	mfd; transcription-repair coupling factor (superfamily II helicase) [EC:3.6.4.-]
K03701	uvrA; excinuclease ABC subunit A
K03702	uvrB; excinuclease ABC subunit B
K03703	uvrC; excinuclease ABC subunit C
K01507	ppa; inorganic pyrophosphatase [EC:3.6.1.1]
K00937	ppk; polyphosphate kinase [EC:2.7.4.1]
K22468	ppk2; polyphosphate kinase [EC:2.7.4.1]
K08682	acpH; acyl carrier protein phosphodiesterase [EC:3.1.4.14]
K00997	acpS; holo-[acyl-carrier protein] synthase [EC:2.7.8.7]
K06153	bacA; undecaprenyl-diphosphatase [EC:3.6.1.27]

K07259	dacB; serine-type D-Ala-D-Ala carboxypeptidase/endopeptidase (penicillin-binding protein 4) [EC:3.4.16.4 3.4.21.-]
K03587	ftsI; cell division protein FtsI (penicillin-binding protein 3) [EC:3.4.16.4]
K05515	mrdA; penicillin-binding protein 2 [EC:3.4.16.4]
K03814	mtgA; monofunctional glycosyltransferase [EC:2.4.1.129]
K05367	pbpC; penicillin-binding protein 1C [EC:2.4.1.129]
K08483	ptsI; phosphoenolpyruvate-protein phosphotransferase (PTS system enzyme I) [EC:2.7.3.9]
K02806	ptsN; nitrogen PTS system EIIA component [EC:2.7.1.-]
K02358	tuf, TUFM; elongation factor Tu
K03594	bfr; bacterioferritin [EC:1.16.3.1]
K07215	pigA, hemO; heme oxygenase (biliverdin-IX-beta and delta-forming) [EC:1.14.99.58]
K03100	lepB; signal peptidase I [EC:3.4.21.89]
K03101	lspA; signal peptidase II [EC:3.4.23.36]
K13993	HSP20; HSP20 family protein
K02594	nifV; homocitrate synthase NifV [EC:2.3.3.14]
K02031	ABC.PE.A; peptide/nickel transport system ATP-binding protein
K02033	ABC.PE.P; peptide/nickel transport system permease protein
K02034	ABC.PE.P1; peptide/nickel transport system permease protein
K02035	ABC.PE.S; peptide/nickel transport system substrate-binding protein
K07711	glrK, qseE; two-component system, NtrC family, sensor histidine kinase GlrK [EC:2.7.13.3]
K07715	glrR, qseF; two-component system, NtrC family, response regulator GlrR
K07667	kdpE; two-component system, OmpR family, KDP operon response regulator KdpE
K07666	qseB; two-component system, OmpR family, response regulator QseB
K07645	qseC; two-component system, OmpR family, sensor histidine kinase QseC [EC:2.7.13.3]
K20266	trbJ; type IV secretion system protein TrbJ
K02871	RP-L13, MRPL13, rplM; large subunit ribosomal protein L13
K02874	RP-L14, MRPL14, rplN; large subunit ribosomal protein L14
K02876	RP-L15, MRPL15, rplO; large subunit ribosomal protein L15
K02878	RP-L16, MRPL16, rplP; large subunit ribosomal protein L16
K02879	RP-L17, MRPL17, rplQ; large subunit ribosomal protein L17
K02881	RP-L18, MRPL18, rplR; large subunit ribosomal protein L18
K02884	RP-L19, MRPL19, rplS; large subunit ribosomal protein L19

K02886	RP-L2, MRPL2, rplB; large subunit ribosomal protein L2
K02887	RP-L20, MRPL20, rplT; large subunit ribosomal protein L20
K02888	RP-L21, MRPL21, rplU; large subunit ribosomal protein L21
K02890	RP-L22, MRPL22, rplV; large subunit ribosomal protein L22
K02892	RP-L23, MRPL23, rplW; large subunit ribosomal protein L23
K02895	RP-L24, MRPL24, rplX; large subunit ribosomal protein L24
K02897	RP-L25, rplY; large subunit ribosomal protein L25
K02899	RP-L27, MRPL27, rpmA; large subunit ribosomal protein L27
K02902	RP-L28, MRPL28, rpmB; large subunit ribosomal protein L28
K02904	RP-L29, rpmC; large subunit ribosomal protein L29
K02906	RP-L3, MRPL3, rplC; large subunit ribosomal protein L3
K02907	RP-L30, MRPL30, rpmD; large subunit ribosomal protein L30
K02909	RP-L31, rpmE; large subunit ribosomal protein L31
K02911	RP-L32, MRPL32, rpmF; large subunit ribosomal protein L32
K02913	RP-L33, MRPL33, rpmG; large subunit ribosomal protein L33
K02914	RP-L34, MRPL34, rpmH; large subunit ribosomal protein L34
K02916	RP-L35, MRPL35, rpmI; large subunit ribosomal protein L35
K02919	RP-L36, MRPL36, rpmJ; large subunit ribosomal protein L36
K02926	RP-L4, MRPL4, rplD; large subunit ribosomal protein L4
K02931	RP-L5, MRPL5, rplE; large subunit ribosomal protein L5
K02933	RP-L6, MRPL6, rplF; large subunit ribosomal protein L6
K02939	RP-L9, MRPL9, rplI; large subunit ribosomal protein L9
K02945	RP-S1, rpsA; small subunit ribosomal protein S1
K02946	RP-S10, MRPS10, rpsJ; small subunit ribosomal protein S10
K02948	RP-S11, MRPS11, rpsK; small subunit ribosomal protein S11
K02952	RP-S13, rpsM; small subunit ribosomal protein S13
K02954	RP-S14, MRPS14, rpsN; small subunit ribosomal protein S14
K02956	RP-S15, MRPS15, rpsO; small subunit ribosomal protein S15
K02959	RP-S16, MRPS16, rpsP; small subunit ribosomal protein S16
K02961	RP-S17, MRPS17, rpsQ; small subunit ribosomal protein S17
K02963	RP-S18, MRPS18, rpsR; small subunit ribosomal protein S18

K02965	RP-S19, rpsS; small subunit ribosomal protein S19
K02967	RP-S2, MRPS2, rpsB; small subunit ribosomal protein S2
K02968	RP-S20, rpsT; small subunit ribosomal protein S20
K02970	RP-S21, MRPS21, rpsU; small subunit ribosomal protein S21
K02982	RP-S3, rpsC; small subunit ribosomal protein S3
K02986	RP-S4, rpsD; small subunit ribosomal protein S4
K02988	RP-S5, MRPS5, rpsE; small subunit ribosomal protein S5
K02990	RP-S6, MRPS6, rpsF; small subunit ribosomal protein S6
K02994	RP-S8, rpsH; small subunit ribosomal protein S8
K02996	RP-S9, MRPS9, rpsI; small subunit ribosomal protein S9
K13288	orn, REX2, REXO2; oligoribonuclease [EC:3.1.-.-]
K03685	rnc, DROSHA, RNT1; ribonuclease III [EC:3.1.26.3]
K05592	deaD, cshA; ATP-dependent RNA helicase DeaD [EC:3.6.4.13]
K08311	nudH; putative (di)nucleoside polyphosphate hydrolase [EC:3.6.1.-]
K00970	pcnB; poly(A) polymerase [EC:2.7.7.19]
K00962	pnp, PNPT1; polyribonucleotide nucleotidyltransferase [EC:2.7.7.8]
K03654	recQ; ATP-dependent DNA helicase RecQ [EC:3.6.4.12]
K11927	rhlE; ATP-dependent RNA helicase RhlE [EC:3.6.4.13]
K03628	rho; transcription termination factor Rho
K08300	rne; ribonuclease E [EC:3.1.26.12]
K12573	rnr, vacB; ribonuclease R [EC:3.1.13.1]
K03040	rpoA; DNA-directed RNA polymerase subunit alpha [EC:2.7.7.6]
K03060	rpoZ; DNA-directed RNA polymerase subunit omega [EC:2.7.7.6]
K00974	cca; tRNA nucleotidyltransferase (CCA-adding enzyme) [EC:2.7.7.72 3.1.3.- 3.1.4.-]
K00384	trxB, TRR; thioredoxin reductase (NADPH) [EC:1.8.1.9]
K01130	E3.1.6.1, aslA; arylsulfatase [EC:3.1.6.1]
K00566	mnmA, trmU; tRNA-uridine 2-sulfurtransferase [EC:2.8.1.13]
K03636	moaD, cysO; sulfur-carrier protein
K21029	moeB; molybdopterin-synthase adenylyltransferase [EC:2.7.7.80]
K03154	thiS; sulfur carrier protein
K04085	tusA, sirA; tRNA 2-thiouridine synthesizing protein A [EC:2.8.1.-]

K09008	NDUFAF3; NADH dehydrogenase [ubiquinone] 1 alpha subcomplex assembly factor 3
K08083	algR; two-component system, LytTR family, response regulator AlgR
K08082	algZ; two-component system, LytTR family, sensor histidine kinase AlgZ [EC:2.7.13.3]
K01644	citE; citrate lyase subunit beta / citryl-CoA lyase [EC:4.1.3.34]
K07663	creB; two-component system, OmpR family, catabolic regulation response regulator CreB
K07641	creC; two-component system, OmpR family, sensor histidine kinase CreC [EC:2.7.13.3]
K07787	cusA, silA; Cu(I)/Ag(I) efflux system membrane protein CusA/SilA
K07798	cusB, silB; membrane fusion protein, Cu(I)/Ag(I) efflux system
K07665	cusR, copR, silR; two-component system, OmpR family, copper resistance phosphate regulon response regulator CusR
K07644	cusS, copS, silS; two-component system, OmpR family, heavy metal sensor histidine kinase CusS [EC:2.7.13.3]
K11103	dctA; aerobic C4-dicarboxylate transport protein
K10125	dctB; two-component system, NtrC family, C4-dicarboxylate transport sensor histidine kinase DctB [EC:2.7.13.3]
K10126	dctD; two-component system, NtrC family, C4-dicarboxylate transport response regulator DctD
K11690	dctM; C4-dicarboxylate transporter, DctM subunit
K11688	dctP; C4-dicarboxylate-binding protein DctP
K11689	dctQ; C4-dicarboxylate transporter, DctQ subunit
K11712	dctR; two-component system, LuxR family, response regulator DctR
K11711	dctS; two-component system, LuxR family, sensor histidine kinase DctS [EC:2.7.13.3]
K04771	degP, htrA; serine protease Do [EC:3.4.21.107]
K01104	E3.1.3.48; protein-tyrosine phosphatase [EC:3.1.3.48]
K07679	evgS, bvgS; two-component system, NarL family, sensor histidine kinase EvgS [EC:2.7.13.3]
K04751	glnB; nitrogen regulatory protein P-II 1
K00990	glnD; [protein-PII] uridylyltransferase [EC:2.7.7.59]
K07712	glnG, ntrC; two-component system, NtrC family, nitrogen regulation response regulator GlnG
K07708	glnL, ntrB; two-component system, NtrC family, nitrogen regulation sensor histidine kinase GlnL [EC:2.7.13.3]
K19641	hupR, hoxA; two-component system, NtrC family, response regulator HupR/HoxA
K19661	hupT, hoxJ; two-component system, NtrC family, sensor histidine kinase HupT/HoxJ [EC:2.7.13.3]
K03620	hyaC; Ni/Fe-hydrogenase 1 B-type cytochrome subunit
K01546	kdpA; potassium-transporting ATPase potassium-binding subunit
K01547	kdpB; potassium-transporting ATPase ATP-binding subunit [EC:7.2.2.6]
K01548	kdpC; potassium-transporting ATPase KdpC subunit

K07646	kdpD; two-component system, OmpR family, sensor histidine kinase KdpD [EC:2.7.13.3]
K07684	narL; two-component system, NarL family, nitrate/nitrite response regulator NarL
K07673	narX; two-component system, NarL family, nitrate/nitrite sensor histidine kinase NarX [EC:2.7.13.3]
K02584	nifA; Nif-specific regulatory protein
K13598	ntrY; two-component system, NtrC family, nitrogen regulation sensor histidine kinase NtrY [EC:2.7.13.3]
K11382	pgtP; MFS transporter, OPA family, phosphoglycerate transporter protein
K07657	phoB; two-component system, OmpR family, phosphate regulon response regulator PhoB
K07636	phoR; two-component system, OmpR family, phosphate regulon sensor histidine kinase PhoR [EC:2.7.13.3]
K02650	pilA; type IV pilus assembly protein PilA
K02667	pilR, pehR; two-component system, NtrC family, response regulator PilR
K02668	pilS, pehS; two-component system, NtrC family, sensor histidine kinase PilS [EC:2.7.13.3]
K15012	regA, regR, actR; two-component system, response regulator RegA
K15011	regB, regS, actS; two-component system, sensor histidine kinase RegB [EC:2.7.13.3]
K07661	rstA; two-component system, OmpR family, response regulator RstA
K07639	rstB; two-component system, OmpR family, sensor histidine kinase RstB [EC:2.7.13.3]
K07774	tctD; two-component system, OmpR family, response regulator TctD
K07649	tctE; two-component system, OmpR family, sensor histidine kinase TctE [EC:2.7.13.3]
K07675	uhpB; two-component system, NarL family, sensor histidine kinase UhpB [EC:2.7.13.3]

References

1.Grasshoff K. 1983. Methods of Seawater Analysis. Acta Hydrochimica et Hydrobiologica 77.

2.Stedmon CA, Bro R. 2008. Characterizing dissolved organic matter fluorescence with parallel factor analysis: a tutorial. Limnology and Oceanography: Methods 6.

3.He W, Hur J. 2015. Conservative behavior of fluorescence EEM-PARAFAC components in resin fractionation processes and its applicability for characterizing dissolved organic matter. Water Research 83:217-226.

4.Kim J, Kim Y, Kang H-W, Kim SH, Rho T, Kang D-J. 2020. Tracing water mass fractions in the deep western Indian Ocean using fluorescent dissolved organic matter. Marine Chemistry 218.

5.Amaral V, Romera-Castillo C, Forja J. 2021. Submarine mud volcanoes as a source of chromophoric dissolved organic matter to the deep waters of the Gulf of Cádiz. Scientific Reports 11.

6.Zhao Y, Song K, Li S, Ma J, Wen Z. 2016. Characterization of CDOM from urban waters in Northern-Northeastern China using excitation-emission matrix fluorescence and parallel factor analysis. Environ Sci Pollut Res Int 23:15381-94.
7.Deutsch C, Weber T. 2012. Nutrient Ratios as a Tracer and Driver of Ocean Biogeochemistry. Annual Review of Marine Science 4:113-141.
8.Kittu LR, Paul AJ, Fernández ‐ Méndez M, Hopwood MJ, Riebesell U. 2023. Coastal N2 Fixation Rates Coincide Spatially With Nitrogen Loss in the Humboldt Upwelling System off Peru. Global Biogeochemical Cycles 37.
9.Gruber N, Sarmiento JL. 1997. Global patterns of marine nitrogen fixation and denitrification. Global Biogeochemical Cycles 11:235-266.
10.Deutsch C, Gruber N, Key RM, Sarmiento JL, Ganachaud A. 2001. Denitrification and N2fixation in the Pacific Ocean. Global Biogeochemical Cycles 15:483-506.
11.Zehr J, McReynolds L. 1989. Use of degenerate oligonucleotides for amplification of the nifH gene from the marine cyanobacterium Trichodesmium thiebautii. Applied and environmental microbiology 55:2522-6.
12.Mo Y, Peng F, Gao X, Xiao P, Logares R, Jeppesen E, Ren K, Xue Y, Yang J. 2021. Low shifts in salinity determined assembly processes and network stability of microeukaryotic plankton communities in a subtropical urban reservoir. Microbiome 9:128.
